# Comparison of Breast Cancer to Healthy Control Tissue Discovers Novel Markers with Potential for Prognosis and Early Detection

**DOI:** 10.1371/journal.pone.0009122

**Published:** 2010-02-09

**Authors:** Michèl Schummer, Ann Green, J. David Beatty, Beth Y. Karlan, Scott Karlan, Jenny Gross, Sean Thornton, Martin McIntosh, Nicole Urban

**Affiliations:** 1 Molecular Diagnostics Program, Fred Hutchinson Cancer Research Center, Seattle, Washington, United States of America; 2 Breast Surgical Clinic, Swedish Cancer Institute, Seattle, Washington, United States of America; 3 Women's Cancer Research Institute at the Samuel Oschin Comprehensive Cancer Institute, Cedars-Sinai Medical Center, Los Angeles, California, United States of America; 4 Saul and Joyce Brandman Breast Center, Cedars-Sinai Medical Center, Los Angeles, California, United States of America; 5 Cellnetix Pathology and Laboratories, Seattle, Washington, United States of America; University Medical Center Hamburg-Eppendorf, Germany

## Abstract

This study was initiated to identify biomarkers with potential value for the early detection of poor-outcome breast cancer. Two sets of well-characterized tissues were utilized: one from breast cancer patients with favorable vs. poor outcome and the other from healthy women undergoing reduction mammaplasty. Over 46 differentially expressed genes were identified from a large list of potential targets by a) mining publicly available expression data (identifying 134 genes for quantitative PCR) and b) utilizing a commercial PCR array. Three genes show elevated expression in cancers with poor outcome and low expression in all other tissues, warranting further investigation as potential blood markers for early detection of cancers with poor outcome. Twelve genes showed lower expression in cancers with poor outcome than in cancers with favorable outcome but no differential expression between aggressive cancers and most healthy controls. These genes are more likely to be useful as prognostic tissue markers than as serum markers for early detection of aggressive disease. As a secondary finding was that, when histologically normal breast tissue was removed from a distant site in a breast with cancer, 7 of 38 specimens displayed a cancer-like expression profile, while the remaining 31 were genetically similar to the reduction mammaplasty control group. This finding suggests that some regions of ipsilateral histologically ‘normal’ breast tissue are predisposed to becoming malignant and that normal-appearing tissue with malignant signature might warrant treatment to prevent new primary tumors.

## Introduction

Our goal in this study was to identify biomarkers with potential value for the early detection of poor-outcome breast cancer. In 1999 we successfully used a transcript-based discovery approach to identify early detection markers for ovarian cancer [1]. Following the 5 phases of screening biomarker development proposed by Pepe *et al.* [2], HE4, the product of the human epididymis gene *WFDC2*, was developed into a serum assay [3], [4] that is now approved for remission monitoring of ovarian cancer. It is being evaluated for its potential role in screening and is considered a successful product of translational biomarker research.

Because breast cancer marker research has focused mainly on prognosis [5], there are few comprehensive studies to identify early detection markers. We therefore relied on our previously successful approach using gene discovery by cDNA microarray followed by expression validation through polymerase chain reaction (PCR), ranking of potential markers and the development and testing of serum assays. For breast cancer, a large body of research already available in the public domain allowed us to forego our own microarray work; instead we mined publicly available expression data in tissues of breast cancer and normal healthy controls.

One of the lessons learned from previous gene discovery experiments is the importance of having high-quality appropriately preserved specimens and matching patient data. We therefore spent considerable effort on the accrual of needed tissues. In close collaboration with participating surgeons and pathologists, we were able to collect specimens in the operating and gross rooms where they were processed with as little delay as possible, thus minimizing variability. In addition, routine clinical gross and microscopic tissue analysis was complemented with routine research histological examination on the actual tissue piece that was later used for expression analysis. Breast tissues from breast cancer patients were then compared to those from healthy individuals. While normal tissue adjacent to the cancer is relatively easy to obtain, we feared that in cancer patients, cancer-related pathways may be perturbed in these tissues [6]. We therefore used normal tissue from breast reduction mammaplasties as controls.

We identified a few genes meeting necessary criteria for potential development as serum markers for diagnosis or early detection of poor-outcome breast cancer. In addition we identified several genes with potential to guide prognosis and therapy.

## Results and Discussion

Our goal was to identify tissue markers that differentiate between healthy breast tissues and breast cancer tissue from patients with poor outcome. This was done in a series of successive steps: Establishment of the range of expression of genes in healthy breast tissue; database mining to identify potential genes with differential expression between breast cancers and controls; expression validation of these genes in breast cancer tissues and controls; and identification of those genes with over-expression in cancers with poor outcome relative to both good-outcome cancer and healthy control tissue.

### Determination of Components of Variation of Normal Tissue

The primary goal was to identify genes that discriminate between normal breast tissue and invasive carcinoma of the breast. Since normal breast tissue was to be used as a reference, a threshold needed to be determined above which a gene would be labeled as differentially expressed. Therefore we assessed the variability in gene expression within normal breast tissue To our knowledge, this has not previously been well studied. Quantitative PCR (SYBR) expression analysis was performed for 18 genes on an average of 3.5 tissue slices per breast from 10 women with bilateral reduction mammaplasty. The PCRs were normalized by their median and the duplicate runs were averaged. Table 1 shows overall gene expression variability (as standard deviation) and which fraction of it is attributable to the component variabilities of woman-to-woman (averaging 64%±9%), within-breast (averaging 30%±9%) and left-to-right breast (averaging 6%±3%). These percentages represent the overall magnitude of the different sources of variation as determined by ANOVA analyses. As expected, between-woman variability is greatest, twice that of within-breast variability, implying that the largest source of variation is heterogeneity among women. Approximately 30% of overall variation was explained by variation in the molecular behavior of different tissue specimens of the same breast (note the assays were performed in duplicate, and the coefficient of variation (CV) of the assay was small compared to each of these components of variation and so can be ignored). The smallest source of variation is the between-breast component, implying that normal material from a contralateral breast is a good surrogate for normal material from the affected breast. Note that the 30% within- and 6% between-breast variation does not imply that two biopsies from different breasts are more similar than two biopsies from the same breast. Rather, informally, a histogram of expression from biopsies across breasts will have a standard deviation that is only 6% larger than the histogram within a breast (the 6% refers to the excess variation when sampling across breasts). Because each value was median-centered, the standard deviation also represents a surrogate for CV in the population; it is scaled to the typical expression levels. Six of the 18 genes have standard deviations above 1 (*COL1A2*, *CTGF*, *GATA3*, *LYZ*, *MUC1* and *WFDC2*) that could be related to a spotty expression pattern (e.g. only a few cells in a tissue express the transcript). Of note, this elevated variability is unrelated to within-breast variability. For *WFDC2*, the gene with the highest variability across all tissue specimens, the standard deviation is 2.73. Therefore, for subsequent comparisons of expression in cancer to that in healthy normal tissue, a threshold will be chosen consisting of the mean expression in healthy breast mammaplasty tissue plus 3 standard deviations (viz. 8.19 for WFDC2). The same calculation will be done for each gene.

**Table 1:** Variability in gene expression.

		Proportion of Standard Deviation		
Marker	StDev	Between women	Within breast	Between breasts (same woman)
**ASPN**	0.88	75%	21%	3%
**CAV1**	0.89	60%	38%	2%
**CFB**	0.74	46%	46%	8%
**COL1A2**	1.13	76%	15%	10%
**CTGF**	1.07	67%	28%	6%
**GATA3**	1.61	60%	36%	4%
**LETMD1**	0.46	79%	11%	9%
**MGST1**	0.94	59%	37%	4%
**LYZ**	1.08	66%	23%	8%
**MMP2**	0.63	50%	39%	10%
**MUC1**	2.34	62%	34%	4%
**SPARC**	0.71	74%	22%	3%
**SUMF2**	0.39	55%	35%	7%
**TIMP1**	0.69	65%	26%	9%
**TIMP2**	0.49	63%	29%	7%
**TIMP3**	0.60	67%	30%	3%
**WFDC2**	2.73	61%	35%	4%
**YWHAZ**	0.37	64%	30%	5%
	**Mean**	**64%**	**30%**	**6%**
	**StDev**	9%	9%	3%

Variability in healthy breast tissue from non-cancer patients measured in 18 genes by ANOVA. The Standard Deviation (StDev) stands for the variability of each individual gene across all tissue specimens. The third column shows the proportion of the standard deviation attributed to differences between women. The fourth column shows the standard deviation attributed to differences within one breast of an individual woman and the last column shows the additional proportion of standard deviation due to the variability between both breasts of the same woman. The overall mean proportion of variability by individual woman is 30% plus 6%.

### Database Mining Identifies Genes with Differential Expression between Breast Cancer and Normal Tissues

Despite a large body of research on gene and protein expression in breast cancer, few studies include healthy controls. In those that do [7], [8], [9], [10], [11], [12], [13], [14], [15], [16], the control tissues are often not well characterized. Most publications report expression only in breast cancer and not in healthy controls. However, these are still useful for a cancer-to-normal comparison since their data can potentially be matched with those from other sources using healthy normal tissues. A sizable number of these breast publications use large-scale analysis, such as microarrays. These and additional expression data were compiled in a database (LevelsDB) that was then mined starting with genes contained in breast cancer data sets (4405 genes), followed by removal of genes and proteins whose subcellular localization makes the protein unlikely to be found in the blood stream by non-necrotic processes (confirmed nuclear, mitochondrial and ribosomal expression), leaving 3271. In a next step, housekeeping genes were excluded, followed by removal of genes with high levels of expression in normal tissues of organs with high cardiac output [17], [18], [19] (expression greater than 10 times the median in bone, brain, heart, kidney, lung, liver, skeletal muscle and pancreas). We rationalize that proteins can potentially be produced at base level in many cells and that big organs (with high cardiac output) may contribute more than others to a basic blood protein level. The higher a basic blood protein level, the harder it is to detect the excess protein made by the developing tumor. From the remaining 1290 genes and proteins we retained those with low expression in normal tissues. The lack of truly normal breast expression data, except for data from one normal tissue coming from a breast with cancer [20] required the use of other normal, especially epithelial tissues for subtractive comparison, contained in six datasets in LevelsDB. The last reduction step resulted in 150 genes and proteins of which 44 were likely to be secreted or membrane bound which makes them ideal as a blood marker. These were augmented by 90 genes for which literature review suggests a potential role as breast cancer markers, resulting in 134 genes (Table 2) for subsequent expression analysis by PCR. References for these additional genes are listed in the supplemental Document S1.

**Table 2:** 71 of 126 genes discriminate cancers from controls.

ADAM12*		CSNK2A1		MIF		SCGB2A1	
AGR2*		**CTGF**	V	**MMP1**	Δ	SCUBE2*	V
**AKT1**		CTHRC1*	Δ	**MMP10**	Δ	**SDC1**	
AMBP*		CYP4B1*	V	**MMP11**	Δ	**SFRP1**	V
ANGPT2*	V	**CYR61**	V	**MMP12**	Δ	SFRP2*	
APOL1*	V	**DEFA1**		**MMP13**	Δ	**SNIP**	
**AR**	V	**DEFA3**		**MMP14**		**SPARC**	V
ASPN*	Δ	ECM1*		**MMP16**		**SPP1**	Δ
BGN*	Δ	**EGFR**	V	**MMP2**	V	STC2*	
**BIRC5**	Δ	**EPO**		**MMP20**		SUMF2*	
**BRCA2**		**EPOR**	V	**MMP3**		**THBS2**	
**BRMS1**		ERBB3*		**MMP7**	V	**TIMP1**	Δ
**BUB1**	Δ	**ERBB4**	V	**MMP8**		**TIMP2**	V
C18orf8*		**ESR1**	Δ	**MMP9**		**TIMP3**	V
**CALB2**		ETAA1*	V	**MSLN**		**TIMP4**	V
**CAV1**	V	FGFR2*	V	**MUC1**	Δ	**TK1**	Δ
**CCNE1**	Δ	FN1*	Δ	**NES**	V	TM9SF2*	
**CD274**	Δ	**FOXA1**	Δ	OAS1*		**TNFRSF10B**	V
**CD44**	V	**GATA3**	Δ	OAS2*	Δ	**TNN**	V
**CDH1**		**GDF15**		**PEBP1**	V	**TOP2A**	Δ
CDKN1B*	V	**HOXB7**	V	**PEBP4**		**TP53**	
**CDX2**		IFIT1*		**PGR**	V	**TRPS1**	
CFB*	Δ	**IGF2**		**PIK3CA**		**TTF1**	
COL11A1*	Δ	**KRT20**		**PIP**		VCAN*	Δ
COL1A1*	Δ	**KRT7**		**PLAUR**	Δ	**VEGFA**	
COL1A2*	Δ	**LCN2**		PRLR*		**VTCN1**	
COL3A1*		**LETMD1**	V	**PROCR**	V	**WFDC2**	Δ
COL5A1*	Δ	**LPAR3**		PSMA5*		**WT1**	Δ
COL5A2*	V	LRRC15*	Δ	PTPN1*		**XBP1**	
COL6A3*		LTF*		**PVRL4**		**YWHAZ**	
COL8A1*		LYZ*		**S100A7**	Δ		
COMP*	Δ	**MGST1**	V	S100B*	V		

126 genes found by mining of expression data and/or LevelsDB (asterisk). Thresholds for cancers and controls were determined by expression in the 28 normal mammaplasty tissues as mean +3 SD for genes with over-expression in the cancers and as below the minimum for genes with under-expression in the cancers. Over- (Δ) or under- (V) expression in cancer tissue with ≥20% of the cancers and ≤5% of the controls above or below threshold.

### Expression Validation Results in 46 Differentially Expressed Genes

To identify from the **134** genes those with the ability to discriminate between normal and malignant breast tissue, PCR expression analysis was performed on 93 tissues (24 invasive cancers, 38 ipsilateral normals, 3 contralateral normals, 28 tissues from breast reduction surgery) from 64 women (Supplemental Tables S1 and S2). The cDNA was oligo-dT primed. A comma-delimited file with the expression data is available in the supplemental document S2. PCR results for 8 genes were not conclusive even after 2 repeats and the genes were removed from further analysis (supplemental Table S3). Of the 126 remaining genes, 67 discriminated between the 25 cancer tissues and the 28 mammaplasty controls with ≥20% of the cancers and ≤5% of the controls above or below threshold (Table 2, and supplemental Table S3).

After completion of the PCR work, a new PCR-based technology (OpenArray by BioTrove, Woburn, MA) had come to the market that allowed for more rapid gene expression analysis [21]. This technology was used to confirm the expression of a subset of the genes on a subset of the tissues. BioTrove's Cancer Pathways OpenArray plate included primer pairs specific for 606 genes associated with DNA repair, angiogenesis, cell adhesion, apoptosis, cell cycle and many genes encoding kinases. Of the 134 genes from database mining, 41 overlapped with the OpenArray panel which was suitable for confirmation of about 30% of the original results. Out of the 94 tissues that were originally tested, 13 were randomly selected from the 24 cancer tissues and likewise 9 from the 28 mammaplasty controls. Applying the same criteria as for the original set, the OpenArray analysis of the reduced set selected the same differentially expressed genes as the analysis of the original set (supplemental Table S4). Both amplifications had good correlation (supplemental Table S5) with an averaged coefficient of variation of 38% (12%–71%), even considering the differences between both methods (oligo-dT versus random priming; differences in primer sequences).

**Figure 1:**
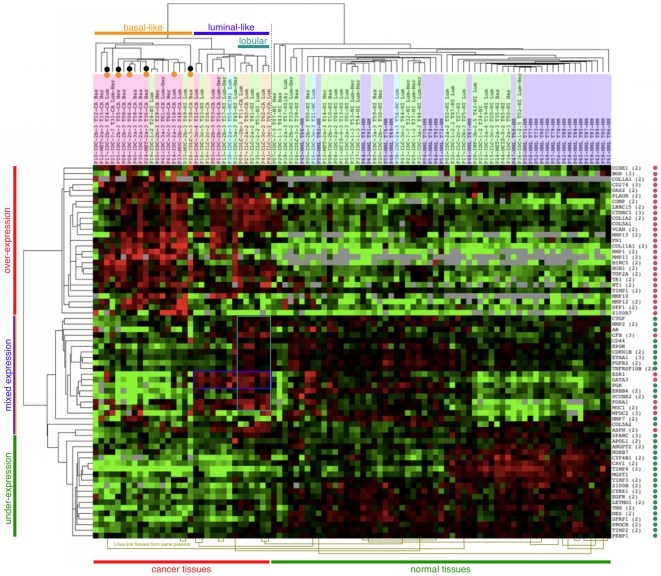
Unsupervised hierarchical clustering of 93 tissues and 67 genes. Unsupervised hierarchical clustering of 93 tissues (24 invasive cancers, 38 ipsilateral normal, 3 contralateral normal, 28 normal tissues from reduction mammaplasty) from 64 patients and 67 genes that discriminate between invasive tissues and mammaplasty normal tissues (red and green dots: over- and under-expression by PCR). Columns: tissues form two distinct clusters (indicated below the figure). Rows: genes form a cancer and a normal cluster, the latter being divided in one with under-expression in all cancer tissues (left, green line) and one with mixed expression (orange-blue line). Luminal-like and basal-like clusters are indicated above the figure. The part of the heat map driving the luminal-like cluster is boxed (blue: luminal-like genes, turquoise: lobular tissues). Tissues from deceased or recurred patients have a black or orange dot above the tissue descriptor which has the following abbreviated components: PatientNo – Diagnosis (IDC = invasive ductal carcinoma, ILC = invasive lobular carcinoma, MET = metaplastic carcinoma, MUC = mucinous carcinoma, NML = normal) – Stage – Grade TissueNo – Description (CA = cancer, NM = normal mammaplasty, NI = normal ipsilateral, NC = normal contralateral) BI-RADS Density Subtype (LUM = luminal, BAS = basal HER2). The tissue descriptors are shaded as follows: orange = lobular cancers, pink = other cancers, green = ipsilateral normals, blue = contralateral normals, purple = mammaplasty normals. Heat map: Red = up-regulation, green = down-regulation, grey = missing or zero value. The lines below the heat map connect tissues from the same patient.

Unsupervised cluster analysis of the 93 tissues and 67 genes shows that 46 genes have the power to separate cancer tissues from controls, thus confirming the validity of our approach (Figure 1, red and green bars in the left). The mammaplasty control patients, seen in Los Angeles, were on average 15 years younger than the patients with tumor or ipsi- and contralateral normal tissue, seen in Seattle. While differences in institution and age could potentially introduce bias, the interspersing of the normal tissues in the control cluster suggests that this is not the case (Figure 1). This can be attributed in part to the strict adherence to identical specimen collection protocols at both sites. No clustering behavior was found based on other factors listed in the supplemental Table S1, including mammography (BI-RADS) score and breast density at the last mammogram before diagnosis, lymph node positivity, tumor size, number of foci, stage and grade.

This first outcome from our analysis was 46 genes that show differential expression between cancers and healthy breast tissues. Having applied a similar strategy to discover serum markers for ovarian cancer ([1], [2], [3], [4]), we have reason to believe that these 46 genes have similar potential because our mining procedure favored proteins with expression outside of the cell and with low potential of being secreted at high level by other healthy tissue. Once proven to be differentially expressed as serum proteins, it will need to be investigated whether a marker could complement mammography in early detection.

### Comparison to Previously Published Results

Comparing the present gene expression results to those previously published is difficult because prior studies rarely used healthy normal controls. While the terms “over-” and “under-expression” are common in the breast cancer literature, they most often refer to expression of one cancer state relative to another or to a cell line, and not relative to a healthy normal control. The three breast cancer publications that used mammaplasty tissue as controls confirm the expression pattern of the metalloproteinases [22], *YWHAZ* [6], *ERBB3* and *ERBB4* [23]. Furthermore *COL1A1* and *COL1A2* over-expression was also seen in a meta-analysis of 13 publications comparing breast cancer to largely undefined and probably ipsilateral normal tissue [24]. This gives credence to the observed expression pattern of the remaining genes.

### Identification of Additional Differentially Expressed Genes

The OpenArray Cancer Pathways chip contained 606 genes of which 41 were used for confirmation of the PCR results. Unsupervised cluster analysis of the remaining 565 genes in the 13 cancers and 9 control tissue results in a clear distinction between over- and under-expressed genes. The OpenArray PCR was not duplicated and thus the results have greater error margins. Therefore more stringent filtering conditions were applied than for the original PCR: genes with differential expression in more than 30% of the tumors at a tumor-to-normal ratio of 1.2 were removed from the dataset. Of the resulting 102 genes, 88% were found to be under-expressed in the tumor tissues (see supplemental Figure S1), as indicated by the negative %CV numbers in Table 3. Once confirmed in their expression, it is likely that some of these 102 genes will be added to the 46 potential markers.

**Table 3:** Genes resulting from the OpenArray analysis.

CSF1	−100%	HADHA	−77%	TP53I3	−62%	SLC2A3	−38%
EGR1	−100%	MCC	−77%	ANPEP	−54%	SMPD1	−38%
FLT1	−100%	RELA	−77%	BAG1	−54%	TGFBI	−38%
FOS	−100%	BTG2	−69%	ILK	−54%	BNIP3	−31%
NID1	−100%	CNBP	−69%	ING1	−54%	CBLB	−31%
SEPP1	−100%	DHX8	−69%	PECAM1	−54%	DEGS1	−31%
SRPX	−100%	EPHA2	−69%	PIR	−54%	EGLN1	−31%
TGFBR2	−100%	GNB2L1	−69%	RIPK1	−54%	ETV6	−31%
TGFBR3	−100%	IGFBP4	−69%	SFRS7	−54%	FOSL2	−31%
TIE1	−100%	NDRG1	−69%	TSG101	−54%	LDHA	−31%
VIM	−100%	PAQR3	−69%	CAPNS1	−46%	NR1D1	−31%
HYAL1	−92%	PEA15	−69%	CHPT1	−46%	PRKCD	−31%
PPARG	−92%	PFDN5	−69%	EIF5	−46%	PRNP	−31%
RAB5A	−92%	RAF1	−69%	GTF2I	−46%	SORT1	−31%
SEMA3C	−92%	RAP1A	−69%	JAK1	−46%	TRADD	−31%
SPRY2	−92%	SKI	−69%	MDM2	−46%	EVL	31%
CCND3	−85%	SP1	−69%	MLLT10	−46%	HSPB1	31%
CDC42BPA	−85%	STK3	−69%	SELENBP1	−46%	KIF3B	38%
CIRBP	−85%	CSF1R	−62%	ATP5B	−38%	PKM2	38%
FOXO1	−85%	GAS6	−62%	AXL	−38%	RFC4	38%
ITGB3	−85%	NF2	−62%	CTNNA1	−38%	RARA	46%
PTEN	−85%	PECI	−62%	DCN	−38%	RAD21	54%
RHOB	−85%	PRKCE	−62%	EXT1	−38%	PRC1	77%
TYRO3	−85%	STAT3	−62%	HRB	−38%	SKIL	77%
ABL1	−77%	TAF1	−62%	PPP2R5A	−38%		
CD59	−77%	TJP1	−62%	PRKD2	−38%		

List of the 102 genes from the OpenArray analysis and percentage of tumor tissues they were differentially expressed in. Negative numbers indicate under-expression.

The high number of under-expressed genes contrasts sharply with above results where over- and under-expressed genes are equally represented. The Cancer Pathways genes had been selected based on general cancer literature which includes a large number of within-cancer and cell line experiments. Our data mining on the other hand focused on breast cancer, normal-to-cancer differences and extracellular expression. Hence, the former contains a larger number of intracellular, regulatory proteins. Interestingly, the differentially expressed genes in both sets are enriched for connective tissue genes, suggesting that alteration in the composition of the connective tissue is an important factor in cancer formation.

### Identification of Markers for Cancers with Poor Outcome

Unsupervised cluster analysis of the original PCR data resulted in a cancer cluster with two sub-clusters, one enriched for patients with cancers of the luminal subtype and one of the basal subtype, as defined by hormone receptor and HER2 expression [25] (Figure 1). Removing the normal tissues from analysis does not alter this clustering behavior (Supplemental Figure S2). The composition of these two sub-clusters is summarized in supplemental Table S6. The luminal-like sub-cluster is defined by over-expression of the luminal markers *ESR1*, *PGR* and *GATA3* [26], [27], [28] in all of its tissues and by the over-expression of *CTGF*, *MMP2*, *AR*, *CFB*, *CD44*, *EPOR*, *CDKN1B*, *ETAA1*, *FGFR2*, *TNFRSF10B*, *ERBB4*, *SCUBE2*, *FOXA1* and *MUC1* in tissues from cancers with lobular or mixed ductal-lobular histology. Except for *AR* [29] and *ESR1* [30], none of these genes can be linked to lobular cancer histology, in particular the comparison by Zhao *et al*. of ductal and lobular carcinomas [31]. The role of *GATA3* for the maintenance of the luminal phenotype has been reviewed by Tlsty, particularly the correlation of low expression of *GATA3* and low estrogen receptor alpha [32] which the present data confirm. The basal-like sub-cluster, characterized by the under-expression of these genes, is enriched for triple-negative (hormone receptor and HER2-negative) cancers and contains all cancer tissues of patients that are deceased (black dots) or have recurred (orange dots). Lobular breast carcinomas are known to be associated with better survival than ductal carcinomas [30], [33] and triple-negative breast cancers have been associated with poor prognosis [34]. Also, in a meta-analysis of published breast cancer cDNA data, low *GATA3* expression is linked with poor clinical outcome [35]. The difference between these cancer sub-clusters could therefore be attributed to the aggressiveness of the disease. Supporting this idea is the fact that the one lobular cancer tissue in the basal-enriched cluster comes from a deceased patient (patient 26), which would infer that severity of outcome supersedes histology.

The genes with over-expression in cancers with favorable as opposed to those with poor outcome are listed in Figure 1 under “mixed expression” on the left. While some of these genes have been linked to the basal subtype [7], [10] and some are now being used to predict disease outcome, including *SCUBE2* in Oncotype DX [36] and Mammaprint [37], the majority of them may constitute a novel group of genes that predict outcome and/or inform treatment. Three genes (S100A7, SPP1 and MMP12) are over-expressed in the cancers with poor outcome compared to both cancers with favorable outcome and healthy controls warranting further investigation as potential blood markers for early detection of cancers with poor outcome.

In summary, the majority of the genes that distinguish cancers by outcome are expressed at lower levels in tissues with poor than in those with favorable outcome. Interestingly, these same genes are also under-expressed in most controls, suggesting that, by and large, molecular profiles might not be able to distinguish aggressive breast cancer from healthy breast tissue. This suggests that these genes have no potential as markers for the detection of aggressive breast cancer, but they hold potential as prognostic markers.

### Histologically Normal Tissues from an Affected Breast Can Demonstrate Molecular Predisposition to Cancer

Unsupervised cluster analysis of the original PCR data placed 7 of the 38 ipsilateral normal tissues in the cancer cluster (Figure 1). The difference between these and the remaining 31 ipsilateral tissues cannot be correlated with tissue or patient characteristics (supplemental Tables S1 and S2). Because BRCA status was not recorded, the study does not address any possible link between mutation and a cancer-like gene expression pattern in ipsilateral normal tissue. Another explanation is a positional effect related to distance between lesion and the site of normal tissue collection or that index lesion and ipsilateral normal tissue come from the same lobe [38]. Consequently, a normal tissue from an unaffected contralateral breast should display a normal-like gene expression pattern. Indeed, of the three contralateral normal tissues, the two coming from a breast without evidence of cancer were found in the normal cluster and the third, from a breast with malignancy, grouped with the cancers.

Tripathi *et al.* compared normal tissue from mammaplasty to ipsilateral normal and breast tissue with *in situ* disease. They found that global gene expression abnormalities exist in both normal epithelium of breast cancer patients and early cancers [6]. The results presented here go one step further by including same-patient invasive tissues. This leads to the conclusion that ipsilateral normal tissues with cancer-like gene expression are molecularly predisposed to cancer. To validate these findings, BRCA status of the patient and positional information of the tissue pieces harvested from a breast would need to be recorded. Our tissue collection protocol has been altered accordingly.

### Conclusions

Our results suggest that many of the genes commonly attributed to cancer pathways are expressed at lower levels in breast cancers than in normal breast tissue, confirming and further extending results by Tripathi *et al.* [6]. Furthermore, the genes that predict aggressive phenotype in between-cancer comparisons are in their majority not differentially expressed between aggressive cancers and healthy controls. If serum assays commonly measure the increase of a marker rather than its absence in cancer, our findings would help explain the current lack of suitable blood markers for breast cancer, particularly in patients with poor prognosis malignancies. With the exception of three genes (*MMP12*, *S100A7* and *SPP1*) whose expression requires further validation, we were unable to find markers with potential utility for the detection of aggressive disease.

In spite of these shortcomings, our work resulted in the identification of a number of differentially expressed genes, including a minimum of 46 discriminating between cancer and controls and 12 related to aggressive disease. These 46 also identify regions of ipsilateral histologically ‘normal’ breast tissue are predisposed to becoming malignant. Of all discovered genes, those coding for proteins that are readily shed may be of interest for serum marker evaluation, assuming a positive correlation between transcript and protein expression and that the proteins are shed into the blood. Markers that are over-expressed but not shed may be the most attractive for tumor-specific localization, including prognosis.

## Methods

### Ethics Statement

This study was conducted according to the principles expressed in the Declaration of Helsinki. The study was approved by the Institutional Review Board of Fred Hutchinson Cancer Research Center (IRB#5317), Cedars Sinai Cancer Center (IRB#4321/CR00002187) and Swedish Medical Center (IRB#3992C-03). All patients provided written informed consent for the collection of samples and subsequent analysis.

### Patients and Tissues

Patients were enrolled at Swedish Medical Center, Seattle and Cedars Sinai Medical Center, Los Angeles. Patients were consented before surgery and administered a health status and family history questionnaire. Hospital records were used for follow-up. Patient characteristics are reported in the supplemental Table S1. Cancer, ipsi- and contralateral normal tissues were obtained at Swedish Medical Center from 44 patients (including 7 with neoadjuvant treatment) undergoing mastectomy. Tissues from breast reduction surgeries (20 patients) were obtained from private practices in Los Angeles and histologically analyzed to exclude any with abnormalities. In all cases, tissue was obtained and processed by research personnel in the operating or gross room and frozen within 1 hour of surgery. The frozen tissue available for research (mean: 150 g, range: 20–500 g) was split into several pieces of which one was fixed in formalin, embedded in paraffin and used for histological examination by a pathologist. The other pieces were kept frozen and used for RNA extraction. No microdissection was performed. Viable tumor cell content in the cancer tissues varied from 15% to 95%, the rest being mostly fibrous and fatty tissue (Supplemental Table S2). We did not select tissues with more than 60% fat content. The variability in tumor cell volume is a consequence of our collection procedure where samples were taken from various distances to the main tumor mass. Furthermore, lobular carcinomas (25% of our cancer tissues) often display low tumor cellularity. Unsupervised cluster analysis of these tissues showed no cluster formation based on tumor cell content (Supplemental Figure S2). In the end, gross and microscopic clinical evaluation matched the histology of the actual tissue piece being analyzed in 50% of the cancer tissues and 67% of tissues with normal histology.

### LevelsDB

Over the last 10 years a database has been compiled (LevelsDB) that holds gene and protein expression information from over 134 publications (90% transcript-, 10% protein-based) and 21,890 genes. LevelsDB was created to facilitate the discovery of markers for cancer detection, and emphasis was given to publications with data for normal controls as well as cancers. LevelsDB uses the GeneID as an identifier [39] which is related to the gene symbols governed by the Guidelines for Human Gene Nomenclature [40]. The datasets were extremely variable in the way they recorded expression, ranging from a simple list of proteins to raw cDNA microarray expression data. As a consequence, LevelsDB forewent exact representation of original expression values. Instead, it recorded whether or not a transcript or protein was present in a given tissue, whether it had tumor-to-normal ratios above a factor 2 or, in the case of cDNA microarray-based expression data, whether a mRNA was expressed at low, medium or high levels (threshold defined by 1x, 3x, and 10x the median expression across all tissues). LevelsDB also contains data on subcellular localization. The datasets used in LevelsDB are listed in the supplemental Document S1. Access to this database is available upon request.

### RNA Extraction and Real-Time PCR (SYBR)

Snap-frozen tissues were homogenized with a TissueLyser (Qiagen, Valencia, CA) in Trizol (Invitrogen, Carlsbad, CA). Total RNA was then extracted using RNeasy with DNAse I (Qiagen). RNA quality was measured by Agilent 2100 Bioanalyzer (Agilent, Santa Clara, CA) to have a 28S/18S RNA ratio of 1±0.2 and by spectrophotometer with an OD_260_/OD_280_ ratio >1.6). Mean total RNA yield was 90±130 µg per mg of tissue. Copy DNA was reverse transcribed from 5 µg of total RNA (Superscript III kit, Invitrogen) with oligo-dT priming, of which 50 ng were used as template in a 15 µl PCR. Copy DNA was amplified using the SYBR green kit (Invitrogen) on a 7900HT Fast Real-Time PCR System (Applied Biosystems, Foster City, CA). Each 384-well plate contained aliquots of all cDNAs used during the experiment as well as a standard made with testes cDNA in 5 dilutions (1∶1, 1∶3, 1∶9, 1∶27, 1∶81) as duplicates, amplified with primers for *ACTB* (all cDNAs were sub-aliquoted and stored at −20°C for consistency). This allowed us to transform the logarithmic cycle threshold (CT) values into linear values. Reactions were performed in duplicate or, if samples did not amplify well or if the correlation between the runs was poor, in triplicate. All PCRs were normalized by the averaged expression of three housekeeping genes *ACTB*, *B2M* and *TMED10* run in triplicate. Primer sequences are listed in the supplemental Document S3.

### OpenArray Transcript Expression

Two micrograms of total RNA were reverse transcribed using the High Capacity cDNA RT Kit (Applied Biosystems) with random hexamer primers. All cDNA was analyzed on the Cancer Pathways OpenArray system (BioTrove, Woburn, MA) using the Fast Start DNA SYBR Green kit (Roche, Nutley, NJ). Four cDNA samples were tested simultaneously per plate, with 16 samples per run. CT values were transformed into linear values by calculating 1.735 ^∧^ (32 - CT). Values were normalized by the mean of 18 housekeeping genes. The expression values from the OpenArray platform and the qPCR (SYBR) were mean-normalized to allow for comparison across the two platforms.

### Cluster Analysis

Unsupervised hierarchical clustering was performed using Spearman rank correlation as similarity metric and centroid linkage as clustering method. PCR expression values were averaged between duplicate runs, mean-normalized and entered into the Cluster program [41] as log2 values. The tree was visualized using Java Treeview [42].

## Supporting Information

Document S1 List of the datasets used in LevelsDB and references for the 134 potential marker genes.
(0.06 MB RTF)

Document S2 PCR expression data of the 134 genes in 93 tissues including explanation of the data processing.
(0.06 MB CSV)

Document S3 Primer sequences for the genes amplified the qPCR.
(0.01 MB CSV)

Table S1 Characteristics of the 64 patients who donated tissue. Age at diagnosis, Diagnosis (IDC = invasive ductal carcinoma, ILC = invasive lobular carcinoma, MET = metaplastic carcinoma, INF = inflammatory carcinoma, MUC = mucinous carcinoma, NML = normal), Stage, grade and tumor size noted during gross histopathological examination, Number of positive lymph nodes out of all tested, BI-RADS mammography score (1 = normal, 2 = benign, 3 =  probably benign, 4 =  suspicious, 5 = malignant), Breast Density score (0 = fatty, 1 = average, 2 = dense, 3 = very dense), Race (White, Black, Hispanic, Asian), Hormone receptor (estrogen and/or progesterone) and ERBB2 status, and the tissue(s) used (NI = normal ipsilateral, CA = invasive cancer, NC = normal contralateral, NM = normal from mammaplasty; fatty = 20-50% fat). Progression-free survival (PFI) and survival given in months. NED = no evidence of disease. Empty cells: no information. It should be noted that the breast reduction population was 15 years younger than the breast cancer population.
(0.47 MB PDF)

Table S2 Tissue Characteristics. Histopathological examination was performed on the tissue piece adjacent to the one used for RNA extraction. Patient numbers in the first column match those in table S1. Tissue classification is explained in table S1. In the case of tumor tissue, the histology lists the percentage of the tissue specimen that was involved by tumor. The percentage of viable tumor cells, necrosis and the inflammatory component within the tumor histology is listed in the last three columns. These numbers donÊ¼t add up to 100% as we did not record the percentage of fibrous or fatty tissue. However, we limited collection of all tissues to those with less than 60% fat. Please note that tumor grade of an individual tissue piece might differ from the grade of the entire tumor listed in table S1.
(0.31 MB PDF)

Table S3 List of the 134 genes (by gene symbol) found by mining of expression data and/or LevelsDB mining (asterisk). References are listed in the Supplement. The 8 genes for which PCR was inconclusive are struck through. Thresholds for the cancers and controls were determined by the expression in the 28 normal mammaplasty tissues as mean +3 SD for genes with over-expression in the cancers and as below the minimum for genes with under-expression in the cancers. These cutoff values lie well above the gene expression variability in normal breast tissues listed in Table 1. Columns 2 and 3 list the percent of cancer or control tissues above or below this threshold. Result column: over- or under-expression in cancer tissue for genes with > = 20% of the cancers and < = 5% of the controls above or below threshold.
(0.06 MB PDF)

Table S4 Comparison of PCR results from the original PCR and the OpenArray results from the reduced tissue and gene set. Listed are the expression values for the tumors (pink and orange shading in the leftmost column) and the mammaplasty normal controls (green shading) for both the original (TOR) and the OpenArray platform (BT). The yellow- and purple-shaded cells represent genes with over- and under-expression respectively. Due to the extreme low number of control tissues (9), values with high error (outliers) have a huge influence on the cutoff and hence the overall results. Four of these outliers were removed (empty cells).
(0.25 MB PDF)

Table S5 Percent CV values of original and unfiltered OpenArray PCR results. Percent CV values for all tissues (%CV a) and for only those tissues with outliers were removed from the OpenArray results (%CV b). Removal was acceptable because the OpenArray PCR was not duplicated like the original PCR and had a higher number of values close or below the PCRÊ¼s detection limit.
(0.04 MB PDF)

Table S6 Composition of the Sub-Cluster of the Cancer Cluster. Shown are the number of patients and the percentage of the total number of patients per cluster. Highlighted are the features that give the patients in the luminal-enriched cluster a better prognosis.
(0.03 MB PDF)

Figure S1 Unsupervised cluster analysis of the filtered OpenArray data (112 genes, 13 invasive cancers and 9 normal control tissues). The OpenArray dataset contains a high proportion of genes with under-expression in the invasive cancer tissues (pink shading on the left) compared to the healthy controls (green shading on the left). The genes found to be under- or over-expressed by the original dataset are indicated by a green or red dot next to their names (right). These are found in the expected cluster, further confirming the original PCR results. The tissue descriptor above lists the histology (IDC in pink, ILC and IDLC in orange and NML in green) followed by the tissue identifier.
(0.23 MB PDF)

Figure S2 Unsupervised cluster analysis of the 24 invasive cancer tissues (7 tissues with > = 60% and 17 with < = 30% viable tumor cells) using the 67 genes that discriminate between invasive tissues and mam- maplasty normal tissues. No cluster formation is observed based on tumor cell content. The two clusters that are formed separate the cancers by outcome.
(0.24 MB PDF)
