# Correction: Comparison of Breast Cancer to Healthy Control Tissue Discovers Novel Markers with Potential for Prognosis and Early Detection

**DOI:** 10.1371/annotation/632c5ae8-271b-4d19-8509-dc3b2eefe6a4

**Published:** 2010-04-02

**Authors:** Michèl Schummer, Ann Green, J. David Beatty, Beth Y. Karlan, Scott Karlan, Jenny Gross, Sean Thornton, Martin McIntosh, Nicole Urban

For patients 22 and 37 the clinical parameters (stage of the disease, grade, and tumor size and, in particular, hormone and HER2 receptor status) are incorrect. Please view the corrected table here:
