# Supplementary material for: Comparison of Breast Cancer to Healthy Control Tissue Discovers Novel Markers with Potential for Prognosis and Early Detection
Source: PLoS One. 2010 Feb 9;5(2):e9122. doi: 10.1371/journal.pone.0009122 (PMC2817747; doi:10.1371/journal.pone.0009122)
Supplement: Document S1 — List of the datasets used in LevelsDB and references for the 134 potential marker genes. (0.06 MB RTF) [file pone.0009122.s001.rtf]

Datasets used in LevelsDBGeneral cancer genes (1-6, 290), Endometrial cancer (7), Head and Neck Cancer (8, 9), Seminoma (10), Thyroid Cancer (11), Breast Cancer (6, 12-25, 285, 286), Cancer of the Central Nervous System (26, 27), Colon Cancer (28-35), Gastric Cancer (36, 37), Renal Cancer (19, 38, 39), Liver Cancer (40-44), Lung Cancer (19, 45-49), Melanoma (3, 50-53), Ovarian Cancer (54-79, 287, 288), Pancreatic Cancer (80-87), Prostate Cancer (56, 88-97), Cell Lines (98, 99, 287), Normal Tissues General (1, 19, 100-103, 287), Normal Breast (104), Normal Blood (105), Normal Brain (26), Normal Colon (28), Normal Pancreas (81), Normal Oral Tissue (9), Normal Thyroid (11), Normal Prostate (106), Normal Kidney (107), Subcellular Localization (98, 108-117, 289).References for the 134 potential marker genesThe following 134 genes were selected by mining of LevelsDB (asterisk) and /or by literature search (references given after the name): ADAM12*, AGR2*, AKT1 (118, 119), AMBP* (120), ANGPT2* (121), APOL1*, AR (122), ASPN*, BGN*, BIRC5 (123, 124), BRCA1 (125, 126), BRCA2 (125), BRMS1 (127, 128), BUB1 (129, 130), C18orf8*, CALB2 (131, 132), CAV1 (133, 134), CCNE1 (135, 136), CD274 (137), CD44 (6, 138, 139), CDH1 (25, 140), CDKN1B* (136, 141), CDX2 (142), CFB*, COL11A1*, COL1A1*, COL1A2*, COL3A1*, COL5A1*, COL5A2* , COL6A3*, COL8A1*, COMP*, CSNK2A1 (143, 144), CTGF (145), CTHRC1*, CXCL1 (146, 147), CYP4B1*, CYR61 (145), DCD*, DEFA1 (148), DEFA3 (148), ECM1*, EGFR (151-154), EPO (155), EPOR (155), ERBB2 (15, 156), ERBB3* , ERBB4 (157), ESR1 (158, 159), ETAA1*, FGFR2* (160, 161), FN1* (162, 163), FOXA1 (164-168), GATA3 (166, 168, 169), GDF15 (170, 171), HOXB7 (22, 172), IFIT1*, IGF2 (173, 174), IL17RB (175), KRT20 (176-178), KRT7 (176-179), LCN2 (180, 181), LETMD1 (182) , LPAR3 (149, 150), LRRC15*, LTF*, LYZ*, MGST1 (183, 184), MIF (185), MMP1 (186-188), MMP10 (187, 189, 190), MMP11 (187, 191-193), MMP12 (187, 189, 193), MMP13 (189, 194), MMP14 (187, 195-197), MMP16 (198, 199), MMP17 (194, 200), MMP2 (201-204), MMP20 (205, 206), MMP3 (195, 207), MMP7 (208-210), MMP8 (188), MMP9 (187, 194, 211), MSLN (212-214), MUC1 (215-217), MYBL2*, NES (218),  OAS1*, OAS2*, PEBP1 (219), PEBP4 (220-222), PGR (223, 224), PIK3CA (225, 226), PIP (227-230), PLAUR (231, 232), PRL (233), PRLR*, PROCR (139, 234), PSMA5*, PTPN1* (235), PVRL4 (236), S100A7 (158, 237), S100B*, SCGB2A1 (238, 239), SCUBE2*, SDC1 (240), SFRP1 (241), SFRP2*, SNIP (242), SPARC (243, 244), SPP1 (245, 246), STC2*, SUMF2*, THBS2 (247-249), TIMP1 (211, 250, 251), TIMP2 (204, 250), TIMP3 (252, 253), TIMP4 (254, 255), TTF1 (256), TK1 (257, 258), TM9SF2*, TNFRSF10B (259, 260), TNN (261, 262), TOP2A (263, 264), TP53 (265-267), TRPS1 (268-270), VCAN* (25, 271), VEGFA (272, 273), VTCN1 (274-277), WFDC2 (278), WT1 (279-281), XBP1 (282, 283), YWHAZ (284).References1.	Ramaswamy S, Tamayo P, Rifkin R, et al. Multiclass cancer diagnosis using tumor gene expression signatures. Proc Natl Acad Sci U S A 2001; 98: 15149-54.2.	Pilarsky C, Wenzig M, Specht T, Saeger HD, Grutzmann R. Identification and validation of commonly overexpressed genes in solid tumors by comparison of microarray data. Neoplasia 2004; 6: 744-50.3.	Covell DG, Wallqvist A, Rabow AA, Thanki N. Molecular classification of cancer: unsupervised self-organizing map analysis of gene expression microarray data. Mol Cancer Ther 2003; 2: 317-32.4.	Chang HY, Sneddon JB, Alizadeh AA, et al. Gene expression signature of fibroblast serum response predicts human cancer progression: similarities between tumors and wounds. PLoS Biol 2004; 2: E7.5.	Glinsky GV, Berezovska O, Glinskii AB. Microarray analysis identifies a death-from-cancer signature predicting therapy failure in patients with multiple types of cancer. J Clin Invest 2005; 115: 1503-21.6.	Liu R, Wang X, Chen GY, et al. The prognostic role of a gene signature from tumorigenic breast-cancer cells. N Engl J Med 2007; 356: 217-26.7.	Risinger JI, Maxwell GL, Chandramouli GV, et al. Microarray analysis reveals distinct gene expression profiles among different histologic types of endometrial cancer. Cancer Res 2003; 63: 6-11.8.	Villaret DB, Wang T, Dillon D, et al. Identification of genes overexpressed in head and neck squamous cell carcinoma using a combination of complementary DNA subtraction and microarray analysis. Laryngoscope 2000; 110: 374-81.9.	Leethanakul C, Knezevic V, Patel V, et al. Gene discovery in oral squamous cell carcinoma through the Head and Neck Cancer Genome Anatomy Project: confirmation by microarray analysis. Oral Oncol 2003; 39: 248-58.10.	Sperger JM, Chen X, Draper JS, et al. Gene expression patterns in human embryonic stem cells and human pluripotent germ cell tumors. Proc Natl Acad Sci U S A 2003; 100: 13350-5.11.	Wasenius VM, Hemmer S, Kettunen E, Knuutila S, Franssila K, Joensuu H. Hepatocyte growth factor receptor, matrix metalloproteinase-11, tissue inhibitor of metalloproteinase-1, and fibronectin are up-regulated in papillary thyroid carcinoma: a cDNA and tissue microarray study. Clin Cancer Res 2003; 9: 68-75.12.	van 't Veer LJ, Dai H, van de Vijver MJ, et al. Gene expression profiling predicts clinical outcome of breast cancer. Nature 2002; 415: 530-6.13.	Perou CM, Sørlie T, Eisen MB, et al. Molecular portraits of human breast tumours. Nature 2000; 406: 747-52.14.	Dressman MA, Walz TM, Lavedan C, et al. Genes that co-cluster with estrogen receptor alpha in microarray analysis of breast biopsies. Pharmacogenomics J 2001; 1: 135-41.15.	Sørlie T, Perou CM, Tibshirani R, et al. Gene expression patterns of breast carcinomas distinguish tumor subclasses with clinical implications. Proc Natl Acad Sci U S A 2001; 98: 10869-74.16.	Sørlie T, Tibshirani R, Parker J, et al. Repeated observation of breast tumor subtypes in independent gene expression data sets. Proc Natl Acad Sci U S A 2003; 100: 8418-23.17.	Zhao H, Langerød A, Ji Y, et al. Different gene expression patterns in invasive lobular and ductal carcinomas of the breast. Mol Biol Cell 2004; 15: 2523-36.18.	Zhu G, Reynolds L, Crnogorac-Jurcevic T, et al. Combination of microdissection and microarray analysis to identify gene expression changes between differentially located tumour cells in breast cancer. Oncogene 2003; 22: 3742-8.19.	Amatschek S, Koenig U, Auer H, et al. Tissue-wide expression profiling using cDNA subtraction and microarrays to identify tumor-specific genes. Cancer Res 2004; 64: 844-56.20.	Abba MC, Drake JA, Hawkins KA, et al. Transcriptomic changes in human breast cancer progression as determined by serial analysis of gene expression. Breast Cancer Res 2004; 6: R499-513.21.	Schneider J, Ruschhaupt M, Buness A, et al. Identification and meta-analysis of a small gene expression signature for the diagnosis of estrogen receptor status in invasive ductal breast cancer. Int J Cancer 2006; 119: 2974-9.22.	Grigoriadis A, Mackay A, Reis-Filho JS, et al. Establishment of the epithelial-specific transcriptome of normal and malignant human breast cells based on MPSS and array expression data. Breast Cancer Res 2006; 8: R56.23.	Naderi A, Teschendorff AE, Barbosa-Morais NL, et al. A gene-expression signature to predict survival in breast cancer across independent data sets. Oncogene 2007; 26: 1507-16.24.	UniGene: Organized View of the Transcriptome. National Center for Biotechnology Information.25.	Turashvili G, Bouchal J, Baumforth K, et al. Novel markers for differentiation of lobular and ductal invasive breast carcinomas by laser microdissection and microarray analysis. BMC Cancer 2007; 7: 55.26.	Pomeroy SL, Tamayo P, Gaasenbeek M, et al. Prediction of central nervous system embryonal tumour outcome based on gene expression. Nature 2002; 415: 436-42.27.	Boon K, Edwards JB, Eberhart CG, Riggins GJ. Identification of astrocytoma associated genes including cell surface markers. BMC Cancer 2004; 4: 39.28.	Bertucci F, Salas S, Eysteries S, et al. Gene expression profiling of colon cancer by DNA microarrays and correlation with histoclinical parameters. Oncogene 2004; 23: 1377-91.29.	Kwon HC, Kim SH, Roh MS, et al. Gene expression profiling in lymph node-positive and lymph node-negative colorectal cancer. Dis Colon Rectum 2004; 47: 141-52.30.	Li M, Lin YM, Hasegawa S, et al. Genes associated with liver metastasis of colon cancer, identified by genome-wide cDNA microarray. Int J Oncol 2004; 24: 305-12.31.	Friedman DB, Hill S, Keller JW, et al. Proteome analysis of human colon cancer by two-dimensional difference gel electrophoresis and mass spectrometry. Proteomics 2004; 4: 793-811.32.	Takemasa I, Higuchi H, Yamamoto H, et al. Construction of preferential cDNA microarray specialized for human colorectal carcinoma: molecular sketch of colorectal cancer. Biochem Biophys Res Commun 2001; 285: 1244-9.33.	Agrawal D, Chen T, Irby R, et al. Osteopontin identified as lead marker of colon cancer progression, using pooled sample expression profiling. J Natl Cancer Inst 2002; 94: 513-21.34.	Alon U, Barkai N, Notterman DA, et al. Broad patterns of gene expression revealed by clustering analysis of tumor and normal colon tissues probed by oligonucleotide arrays. Proc Natl Acad Sci U S A 1999; 96: 6745-50.35.	Notterman DA, Alon U, Sierk AJ, Levine AJ. Transcriptional gene expression profiles of colorectal adenoma, adenocarcinoma, and normal tissue examined by oligonucleotide arrays. Cancer Res 2001; 61: 3124-30.36.	Chen X, Leung SY, Yuen ST, et al. Variation in gene expression patterns in human gastric cancers. Mol Biol Cell 2003; 14: 3208-15.37.	Inoue H, Matsuyama A, Mimori K, Ueo H, Mori M. Prognostic score of gastric cancer determined by cDNA microarray. Clin Cancer Res 2002; 8: 3475-9.38.	Higgins JP, Shinghal R, Gill H, et al. Gene expression patterns in renal cell carcinoma assessed by complementary DNA microarray. Am J Pathol 2003; 162: 925-32.39.	Li CM, Guo M, Borczuk A, et al. Gene expression in Wilms' tumor mimics the earliest committed stage in the metanephric mesenchymal-epithelial transition. Am J Pathol 2002; 160: 2181-90.40.	Chung EJ, Sung YK, Farooq M, et al. Gene expression profile analysis in human hepatocellular carcinoma by cDNA microarray. Mol Cells 2002; 14: 382-7.41.	Okabe H, Satoh S, Kato T, et al. Genome-wide analysis of gene expression in human hepatocellular carcinomas using cDNA microarray: identification of genes involved in viral carcinogenesis and tumor progression. Cancer Res 2001; 61: 2129-37.42.	Xu XR, Huang J, Xu ZG, et al. Insight into hepatocellular carcinogenesis at transcriptome level by comparing gene expression profiles of hepatocellular carcinoma with those of corresponding noncancerous liver. Proc Natl Acad Sci U S A 2001; 98: 15089-94.43.	Xu L, Hui L, Wang S, et al. Expression profiling suggested a regulatory role of liver-enriched transcription factors in human hepatocellular carcinoma. Cancer Res 2001; 61: 3176-81.44.	Li Y, Tang R, Xu H, et al. Discovery and analysis of hepatocellular carcinoma genes using cDNA microarrays. J Cancer Res Clin Oncol 2002; 128: 369-79.45.	Bangur CS, Switzer A, Fan L, Marton MJ, Meyer MR, Wang T. Identification of genes over-expressed in small cell lung carcinoma using suppression subtractive hybridization and cDNA microarray expression analysis. Oncogene 2002; 21: 3814-25.46.	Wang T, Hopkins D, Schmidt C, et al. Identification of genes differentially over-expressed in lung squamous cell carcinoma using combination of cDNA subtraction and microarray analysis. Oncogene 2000; 19: 1519-28.47.	Chen G, Gharib TG, Huang CC, et al. Proteomic analysis of lung adenocarcinoma: identification of a highly expressed set of proteins in tumors. Clin Cancer Res 2002; 8: 2298-305.48.	Bhattacharjee A, Richards WG, Staunton J, et al. Classification of human lung carcinomas by mRNA expression profiling reveals distinct adenocarcinoma subclasses. Proc Natl Acad Sci U S A 2001; 98: 13790-5.49.	Wigle DA, Jurisica I, Radulovich N, et al. Molecular profiling of non-small cell lung cancer and correlation with disease-free survival. Cancer Res 2002; 62: 3005-8.50.	Zuidervaart W, van der Velden PA, Hurks MH, et al. Gene expression profiling identifies tumour markers potentially playing a role in uveal melanoma development. Br J Cancer 2003; 89: 1914-9.51.	Bittner M, Meltzer P, Chen Y, et al. Molecular classification of cutaneous malignant melanoma by gene expression profiling. Nature 2000; 406: 536-40.52.	Seykora JT, Jih D, Elenitsas R, Horng WH, Elder DE. Gene expression profiling of melanocytic lesions. Am J Dermatopathol 2003; 25: 6-11.53.	Hoek K, Rimm DL, Williams KR, et al. Expression profiling reveals novel pathways in the transformation of melanocytes to melanomas. Cancer Res 2004; 64: 5270-82.54.	Schummer M, Ng W, Bumgarner R, et al. Comparative hybridization of an array of 21,500 ovarian cDNAs for the discovery of genes overexpressed in ovarian carcinomas. Gene 1999; 238: 375-85.55.	Wong KK, Cheng RS, Mok SC. Identification of differentially expressed genes from ovarian cancer cells by MICROMAX cDNA microarray system. Biotechniques 2001; 30: 670-5.56.	Lash AE, Tolstoshev CM, Wagner L, et al. SAGEmap: a public gene expression resource. Genome Res 2000; 10: 1051-60.57.	Yang SH, Kim JS, Oh TJ, et al. Genome-scale analysis of resveratrol-induced gene expression profile in human ovarian cancer cells using a cDNA microarray. Int J Oncol 2003; 22: 741-50.58.	Kachman MT, Wang H, Schwartz DR, Cho KR, Lubman DM. A 2-D liquid separations/mass mapping method for interlysate comparison of ovarian cancers. Anal Chem 2002; 74: 1779-91.59.	Adib TR, Henderson S, Perrett C, et al. Predicting biomarkers for ovarian cancer using gene-expression microarrays. Br J Cancer 2004; 90: 686-92.60.	Ono K, Tanaka T, Tsunoda T, et al. Identification by cDNA microarray of genes involved in ovarian carcinogenesis. Cancer Res 2000; 60: 5007-11.61.	Welsh JB, Zarrinkar PP, Sapinoso LM, et al. Analysis of gene expression profiles in normal and neoplastic ovarian tissue samples identifies candidate molecular markers of epithelial ovarian cancer. Proceedings of the National Academy of Sciences of the United States of America 2001; 98: 1176-81.62.	Wang K, Gan L, Jeffery E, et al. Monitoring gene expression profile changes in ovarian carcinomas using cDNA microarray. Gene 1999; 229: 101-8.63.	Lu KH, Patterson AP, Wang L, et al. Selection of potential markers for epithelial ovarian cancer with gene expression arrays and recursive descent partition analysis. Clin Cancer Res 2004; 10: 3291-300.64.	Heinzelmann-Schwarz VA, Gardiner-Garden M, Henshall SM, et al. Overexpression of the cell adhesion molecules DDR1, Claudin 3, and Ep-CAM in metaplastic ovarian epithelium and ovarian cancer. Clin Cancer Res 2004; 10: 4427-36.65.	Hibbs K, Skubitz K, Pambuccian S, et al. Differential gene expression in ovarian carcinoma: identification of potential biomarkers. Am J Pathol 2004; 165: 397-414.66.	He QY, Zhou Y, Wong E, et al. Proteomic analysis of a preneoplastic phenotype in ovarian surface epithelial cells derived from prophylactic oophorectomies. Gynecol Oncol 2005; 98: 68-76.67.	Bignotti E, Tassi RA, Calza S, et al. Differential gene expression profiles between tumor biopsies and short-term primary cultures of ovarian serous carcinomas: identification of novel molecular biomarkers for early diagnosis and therapy. Gynecol Oncol 2006; 103: 405-16.68.	Buckanovich RJ, Sasaroli D, O'Brien-Jenkins A, et al. Tumor vascular proteins as biomarkers in ovarian cancer. J Clin Oncol 2007; 25: 852-61.69.	Liu J, Yang G, Thompson-Lanza JA, et al. A genetically defined model for human ovarian cancer. Cancer Res 2004; 64: 1655-63.70.	Macleod K, Mullen P, Sewell J, et al. Altered ErbB receptor signaling and gene expression in cisplatin-resistant ovarian cancer. Cancer Res 2005; 65: 6789-800.71.	Vekris A, Meynard D, Haaz MC, Bayssas M, Bonnet J, Robert J. Molecular determinants of the cytotoxicity of platinum compounds: the contribution of in silico research. Cancer Res 2004; 64: 356-62.72.	Roberts D, Schick J, Conway S, et al. Identification of genes associated with platinum drug sensitivity and resistance in human ovarian cancer cells. Br J Cancer 2005; 92: 1149-58.73.	Jazaeri A, Yee C, Sotiriou C, Brantley K, Boyd J, Liu E. Gene expression profiles of BRCA1-linked, BRCA2-linked, and sporadic ovarian cancers. Journal of the National Cancer Institute 2002; 94: 990-1000.74.	Berchuck A, Iversen ES, Lancaster JM, et al. Patterns of gene expression that characterize long-term survival in advanced stage serous ovarian cancers. Clin Cancer Res 2005; 11: 3686-96.75.	Lancaster JM, Dressman HK, Whitaker RS, et al. Gene expression patterns that characterize advanced stage serous ovarian cancers. J Soc Gynecol Investig 2004; 11: 51-9.76.	Spentzos D, Levine DA, Ramoni MF, et al. Gene expression signature with independent prognostic significance in epithelial ovarian cancer. J Clin Oncol 2004; 22: 4700-10.77.	Spentzos D, Levine DA, Kolia S, et al. Unique gene expression profile based on pathologic response in epithelial ovarian cancer. J Clin Oncol 2005; 23: 7911-8.78.	Helleman J, Jansen MPHM, Span PN, et al. Molecular profiling of platinum resistant ovarian cancer. International Journal of Cancer 2006; 118: 1963-71.79.	Schaner ME, Ross DT, Ciaravino G, et al. Gene expression patterns in ovarian carcinomas. Mol Biol Cell 2003; 14: 4376-86.80.	Tan ZJ, Hu XG, Cao GS, Tang Y. Analysis of gene expression profile of pancreatic carcinoma using cDNA microarray. World J Gastroenterol 2003; 9: 818-23.81.	Iacobuzio-Donahue CA, Maitra A, Shen-Ong GL, et al. Discovery of novel tumor markers of pancreatic cancer using global gene expression technology. Am J Pathol 2002; 160: 1239-49.82.	Iacobuzio-Donahue CA, Maitra A, Olsen M, et al. Exploration of global gene expression patterns in pancreatic adenocarcinoma using cDNA microarrays. Am J Pathol 2003; 162: 1151-62.83.	Yu XJ, Long J, Fu DL, Zhang QH, Ni QX. Analysis of gene expression profiles in pancreatic carcinoma by using cDNA microarray. Hepatobiliary Pancreat Dis Int 2003; 2: 467-70.84.	Logsdon CD, Simeone DM, Binkley C, et al. Molecular profiling of pancreatic adenocarcinoma and chronic pancreatitis identifies multiple genes differentially regulated in pancreatic cancer. Cancer Res 2003; 63: 2649-57.85.	Fukushima N, Sato N, Prasad N, Leach SD, Hruban RH, Goggins M. Characterization of gene expression in mucinous cystic neoplasms of the pancreas using oligonucleotide microarrays. Oncogene 2004; 23: 9042-51.86.	Crnogorac-Jurcevic T, Missiaglia E, Blaveri E, et al. Molecular alterations in pancreatic carcinoma: expression profiling shows that dysregulated expression of S100 genes is highly prevalent. J Pathol 2003; 201: 63-74.87.	Grutzmann R, Boriss H, Ammerpohl O, et al. Meta-analysis of microarray data on pancreatic cancer defines a set of commonly dysregulated genes. Oncogene 2005; 24: 5079-88.88.	Bull JH, Ellison G, Patel A, et al. Identification of potential diagnostic markers of prostate cancer and prostatic intraepithelial neoplasia using cDNA microarray. Br J Cancer 2001; 84: 1512-9.89.	DePrimo SE, Diehn M, Nelson JB, et al. Transcriptional programs activated by exposure of human prostate cancer cells to androgen. Genome Biol 2002; 3: RESEARCH0032.90.	Lapointe J, Li C, Higgins JP, et al. Gene expression profiling identifies clinically relevant subtypes of prostate cancer. Proc Natl Acad Sci USA 2004; 101: 811-6.91.	Zhao H, Whitfield ML, Xu T, Botstein D, Brooks JD. Diverse effects of methylseleninic acid on the transcriptional program of human prostate cancer cells. Mol Biol Cell 2004; 15: 506-19.92.	Wright ME, Eng J, Sherman J, et al. Identification of androgen-coregulated protein networks from the microsomes of human prostate cancer cells. Genome Biol 2003; 5: R4.93.	Martin DB, Gifford DR, Wright ME, et al. Quantitative proteomic analysis of proteins released by neoplastic prostate epithelium. Cancer Res 2004; 64: 347-55.94.	Wright ME, Tsai MJ, Aebersold R. Androgen receptor represses the neuroendocrine transdifferentiation process in prostate cancer cells. Mol Endocrinol 2003; 17: 1726-37.95.	Singh D, Febbo PG, Ross K, et al. Gene expression correlates of clinical prostate cancer behavior. Cancer Cell 2002; 1: 203-9.96.	Kristiansen G, Pilarsky C, Wissmann C, et al. Expression profiling of microdissected matched prostate cancer samples reveals CD166/MEMD and CD24 as new prognostic markers for patient survival. J Pathol 2005; 205: 359-76.97.	Meehan KL, Sadar MD. Quantitative profiling of LNCaP prostate cancer cells using isotope-coded affinity tags and mass spectrometry. Proteomics 2004; 4: 1116-34.98.	Faça VM, Ventura AP, Fitzgibbon MP, et al. Proteomic analysis of ovarian cancer cells reveals dynamic processes of protein secretion and shedding of extra-cellular domains. PLoS ONE 2008; 3: e2425.99.	Ross DT, Scherf U, Eisen MB, et al. Systematic variation in gene expression patterns in human cancer cell lines. Nat Genet 2000; 24: 227-35.100.	Khan J. Oncogenomics.  Pediatric Oncology Branch: National Cancer Institute; 2003.101.	Gene Expression Atlas: Text Query. December 2000 ed: Genomics Institute of the Novartis Research Foundation; 2007.102.	BioGPS. 2008 ed: Genomics Institute of the Novartis Research Foundation.103.	Shyamsundar R, Kim YH, Higgins JP, et al. A DNA microarray survey of gene expression in normal human tissues. Genome Biol 2005; 6: R22.104.	Jones C, Mackay A, Grigoriadis A, et al. Expression profiling of purified normal human luminal and myoepithelial breast cells: identification of novel prognostic markers for breast cancer. Cancer Res 2004; 64: 3037-45.105.	Whitney AR, Diehn M, Popper SJ, et al. Individuality and variation in gene expression patterns in human blood. Proc Natl Acad Sci U S A 2003; 100: 1896-901.106.	Clegg N, Abbott D, Ferguson C, Coleman R, Nelson PS. Characterization and comparative analyses of transcriptomes from the normal and neoplastic human prostate. Prostate 2004; 60: 227-39.107.	Higgins JP, Wang L, Kambham N, et al. Gene expression in the normal adult human kidney assessed by complementary DNA microarray. Mol Biol Cell 2004; 15: 649-56.108.	Diehn M, Eisen MB, Botstein D, Brown PO. Large-scale identification of secreted and membrane-associated gene products using DNA microarrays. Nat Genet 2000; 25: 58-62.109.	Shin BK, Wang H, Yim AM, et al. Global profiling of the cell surface proteome of cancer cells uncovers an abundance of proteins with chaperone function. J Biol Chem 2003; 278: 7607-16.110.	Adam PJ, Boyd R, Tyson KL, et al. Comprehensive proteomic analysis of breast cancer cell membranes reveals unique proteins with potential roles in clinical cancer. J Biol Chem 2003; 278: 6482-9.111.	Peirce MJ, Wait R, Begum S, Saklatvala J, Cope AP. Expression profiling of lymphocyte plasma membrane proteins. Mol Cell Proteomics 2004; 3: 56-65.112.	Clark HF, Gurney AL, Abaya E, et al. The secreted protein discovery initiative (SPDI), a large-scale effort to identify novel human secreted and transmembrane proteins: a bioinformatics assessment. Genome Res 2003; 13: 2265-70.113.	Protein Reviews On The Web; Index of information available from PROW. National Cancer Institute.114.	Haverty PM, Weng Z, Best NL, et al. HugeIndex: a database with visualization tools for high-density oligonucleotide array data from normal human tissues. Nucleic Acids Res 2002; 30: 214-7.115.	Hsiao LL, Dangond F, Yoshida T, et al. A compendium of gene expression in normal human tissues. Physiol Genomics 2001; 7: 97-104.116.	Stitziel NO, Mar BG, Liang J, Westbrook CA. Membrane-associated and secreted genes in breast cancer. Cancer Res 2004; 64: 8682-7.117.	Diehn M, Bhattacharya R, Botstein D, Brown PO. Genome-scale identification of membrane-associated human mRNAs. PLoS Genet 2006; 2: e11.118.	Ju X, Katiyar S, Wang C, et al. Akt1 governs breast cancer progression in vivo. Proc Natl Acad Sci U S A 2007; 104: 7438-43.119.	Yoon SY, Kim JM, Oh JH, et al. Gene expression profiling of human HBV- and/or HCV-associated hepatocellular carcinoma cells using expressed sequence tags. Int J Oncol 2006; 29: 315-27.120.	Hamm A, Veeck J, Bektas N, et al. Frequent expression loss of Inter-alpha-trypsin inhibitor heavy chain (ITIH) genes in multiple human solid tumors: a systematic expression analysis. BMC Cancer 2008; 8: 25.121.	Tsutsui S, Inoue H, Yasuda K, et al. Angiopoietin 2 expression in invasive ductal carcinoma of the breast: its relationship to the VEGF expression and microvessel density. Breast Cancer Res Treat 2006; 98: 261-6.122.	Li AJ, Scoles DR, Armstrong KU, Karlan BY. Androgen receptor cytosine-adenine-guanine repeat polymorphisms modulate EGFR signaling in epithelial ovarian carcinomas. Gynecol Oncol 2008; 109: 220-5.123.	Hinnis AR, Luckett JC, Walker RA. Survivin is an independent predictor of short-term survival in poor prognostic breast cancer patients. Br J Cancer 2007; 96: 639-45.124.	Yamashita S, Masuda Y, Kurizaki T, et al. Survivin expression predicts early recurrence in early-stage breast cancer. Anticancer Res 2007; 27: 2803-8.125.	Malone KE, Daling JR, Neal C, et al. Frequency of BRCA1/BRCA2 mutations in a population-based sample of young breast carcinoma cases. Cancer 2000; 88: 1393-402.126.	Rajan JV, Wang M, Marquis ST, Chodosh LA. Brca2 is coordinately regulated with Brca1 during proliferation and differentiation in mammary epithelial cells. Proc Natl Acad Sci U S A 1996; 93: 13078-83.127.	Lombardi G, Di Cristofano C, Capodanno A, et al. High level of messenger RNA for BRMS1 in primary breast carcinomas is associated with poor prognosis. Int J Cancer 2007; 120: 1169-78.128.	Hurst DR, Xie Y, Edmonds MD, Welch DR. Multiple forms of BRMS1 are differentially expressed in the MCF10 isogenic breast cancer progression model. Clin Exp Metastasis 2008.129.	Scintu M, Vitale R, Prencipe M, et al. Genomic instability and increased expression of BUB1B and MAD2L1 genes in ductal breast carcinoma. Cancer Lett 2007; 254: 298-307.130.	Cahill DP, Lengauer C, Yu J, et al. Mutations of mitotic checkpoint genes in human cancers. Nature 1998; 392: 300-3.131.	DeVilliers P, Liu H, Suggs C, et al. Calretinin expression in the differential diagnosis of human ameloblastoma and keratocystic odontogenic tumor. Am J Surg Pathol 2008; 32: 256-60.132.	Vonlanthen S, Kawecki TJ, Betticher DC, Pfefferli M, Schwaller B. Heterozygosity of SNP513 in intron 9 of the human calretinin gene (CALB2) is a risk factor for colon cancer. Anticancer Res 2007; 27: 4279-88.133.	Liedtke C, Kersting C, Burger H, Kiesel L, Wulfing P. Caveolin-1 expression in benign and malignant lesions of the breast. World J Surg Oncol 2007; 5: 110.134.	Elsheikh SE, Green AR, Rakha EA, et al. Caveolin 1 and Caveolin 2 are associated with breast cancer basal-like and triple-negative immunophenotype. Br J Cancer 2008; 99: 327-34.135.	Hunt KK, Keyomarsi K. Cyclin E as a prognostic and predictive marker in breast cancer. Semin Cancer Biol 2005; 15: 319-26.136.	Porter PL, Barlow WE, Yeh IT, et al. p27(Kip1) and cyclin E expression and breast cancer survival after treatment with adjuvant chemotherapy. J Natl Cancer Inst 2006; 98: 1723-31.137.	Ghebeh H, Tulbah A, Mohammed S, et al. Expression of B7-H1 in breast cancer patients is strongly associated with high proliferative Ki-67-expressing tumor cells. Int J Cancer 2007; 121: 751-8.138.	Neve RM, Chin K, Fridlyand J, et al. A collection of breast cancer cell lines for the study of functionally distinct cancer subtypes. Cancer Cell 2006; 10: 515-27.139.	Shipitsin M, Campbell LL, Argani P, et al. Molecular definition of breast tumor heterogeneity. Cancer Cell 2007; 11: 259-73.140.	Bertucci F, Orsetti B, Negre V, et al. Lobular and ductal carcinomas of the breast have distinct genomic and expression profiles. Oncogene 2008; 27: 5359-72.141.	Williamson EA, Wolf I, O'Kelly J, Bose S, Tanosaki S, Koeffler HP. BRCA1 and FOXA1 proteins coregulate the expression of the cell cycle-dependent kinase inhibitor p27(Kip1). Oncogene 2006; 25: 1391-9.142.	Park SY, Kim BH, Kim JH, Lee S, Kang GH. Panels of immunohistochemical markers help determine primary sites of metastatic adenocarcinoma. Arch Pathol Lab Med 2007; 131: 1561-7.143.	Munstermann U, Fritz G, Seitz G, Lu YP, Schneider HR, Issinger OG. Casein kinase II is elevated in solid human tumours and rapidly proliferating non-neoplastic tissue. Eur J Biochem 1990; 189: 251-7.144.	O-charoenrat P, Rusch V, Talbot SG, et al. Casein kinase II alpha subunit and C1-inhibitor are independent predictors of outcome in patients with squamous cell carcinoma of the lung. Clin Cancer Res 2004; 10: 5792-803.145.	Jiang WG, Watkins G, Fodstad O, Douglas-Jones A, Mokbel K, Mansel RE. Differential expression of the CCN family members Cyr61, CTGF and Nov in human breast cancer. Endocr Relat Cancer 2004; 11: 781-91.146.	Hu Y, Sun H, Drake J, et al. From mice to humans: identification of commonly deregulated genes in mammary cancer via comparative SAGE studies. Cancer Res 2004; 64: 7748-55.147.	Bieche I, Chavey C, Andrieu C, et al. CXC chemokines located in the 4q21 region are up-regulated in breast cancer. Endocr Relat Cancer 2007; 14: 1039-52.148.	Li J, Zhao J, Yu X, et al. Identification of biomarkers for breast cancer in nipple aspiration and ductal lavage fluid. Clin Cancer Res 2005; 11: 8312-20.149.	Mills GB, Eder A, Fang X, et al. Critical role of lysophospholipids in the pathophysiology, diagnosis, and management of ovarian cancer. Cancer Treat Res 2002; 107: 259-83.150.	Chou CH, Wei LH, Kuo ML, et al. Up-regulation of interleukin-6 in human ovarian cancer cell via a Gi/PI3K-Akt/NF-kappaB pathway by lysophosphatidic acid, an ovarian cancer-activating factor. Carcinogenesis 2005; 26: 45-52.151.	Bhargava R, Gerald WL, Li AR, et al. EGFR gene amplification in breast cancer: correlation with epidermal growth factor receptor mRNA and protein expression and HER-2 status and absence of EGFR-activating mutations. Mod Pathol 2005; 18: 1027-33.152.	Lebeau A, Unholzer A, Amann G, et al. EGFR, HER-2/neu, cyclin D1, p21 and p53 in correlation to cell proliferation and steroid hormone receptor status in ductal carcinoma in situ of the breast. Breast Cancer Res Treat 2003; 79: 187-98.153.	DiGiovanna MP, Stern DF, Edgerton SM, Whalen SG, Moore D, 2nd, Thor AD. Relationship of epidermal growth factor receptor expression to ErbB-2 signaling activity and prognosis in breast cancer patients. J Clin Oncol 2005; 23: 1152-60.154.	Forrest AR, Taylor DF, Crowe ML, et al. Genome-wide review of transcriptional complexity in mouse protein kinases and phosphatases. Genome Biol 2006; 7: R5.155.	Acs G, Acs P, Beckwith SM, et al. Erythropoietin and Erythropoietin Receptor Expression in Human Cancer. Cancer Res 2001; 61: 3561-5.156.	Bertucci F, Finetti P, Cervera N, et al. Gene expression profiling shows medullary breast cancer is a subgroup of basal breast cancers. Cancer Res 2006; 66: 4636-44.157.	Junttila TT, Sundvall M, Lundin M, et al. Cleavable ErbB4 isoform in estrogen receptor-regulated growth of breast cancer cells. Cancer Res 2005; 65: 1384-93.158.	Smalley MJ, Iravani M, Leao M, et al. Regulator of G-protein signalling 2 mRNA is differentially expressed in mammary epithelial subpopulations and over-expressed in the majority of breast cancers. Breast Cancer Res 2007; 9: R85.159.	Duss S, Andre S, Nicoulaz AL, et al. An oestrogen-dependent model of breast cancer created by transformation of normal human mammary epithelial cells. Breast Cancer Res 2007; 9: R38.160.	Tozlu S, Girault I, Vacher S, et al. Identification of novel genes that co-cluster with estrogen receptor alpha in breast tumor biopsy specimens, using a large-scale real-time reverse transcription-PCR approach. Endocr Relat Cancer 2006; 13: 1109-20.161.	Hunter DJ, Kraft P, Jacobs KB, et al. A genome-wide association study identifies alleles in FGFR2 associated with risk of sporadic postmenopausal breast cancer. Nat Genet 2007; 39: 870-4.162.	Williams CM, Engler AJ, Slone RD, Galante LL, Schwarzbauer JE. Fibronectin expression modulates mammary epithelial cell proliferation during acinar differentiation. Cancer Res 2008; 68: 3185-92.163.	Venables JP, Klinck R, Bramard A, et al. Identification of alternative splicing markers for breast cancer. Cancer Res 2008; 68: 9525-31.164.	Habashy HO, Powe DG, Rakha EA, et al. Forkhead-box A1 (FOXA1) expression in breast cancer and its prognostic significance. Eur J Cancer 2008; 44: 1541-51.165.	Badve S, Turbin D, Thorat MA, et al. FOXA1 expression in breast cancer--correlation with luminal subtype A and survival. Clin Cancer Res 2007; 13: 4415-21.166.	Wilson BJ, Giguere V. Meta-analysis of human cancer microarrays reveals GATA3 is integral to the estrogen receptor alpha pathway. Mol Cancer 2008; 7: 49.167.	Thorat MA, Marchio C, Morimiya A, et al. Forkhead box A1 expression in breast cancer is associated with luminal subtype and good prognosis. J Clin Pathol 2008; 61: 327-32.168.	Eeckhoute J, Keeton EK, Lupien M, Krum SA, Carroll JS, Brown M. Positive cross-regulatory loop ties GATA-3 to estrogen receptor alpha expression in breast cancer. Cancer Res 2007; 67: 6477-83.169.	Kouros-Mehr H, Bechis SK, Slorach EM, et al. GATA-3 links tumor differentiation and dissemination in a luminal breast cancer model. Cancer Cell 2008; 13: 141-52.170.	Welsh JB, Sapinoso LM, Kern SG, et al. Large-scale delineation of secreted protein biomarkers overexpressed in cancer tissue and serum. Proc Natl Acad Sci U S A 2003; 100: 3410-5.171.	Kim JS, Lee C, Bonifant CL, Ressom H, Waldman T. Activation of p53-dependent growth suppression in human cells by mutations in PTEN or PIK3CA. Mol Cell Biol 2007; 27: 662-77.172.	Naora H, Yang YQ, Montz FJ, Seidman JD, Kurman RJ, Roden RB. A serologically identified tumor antigen encoded by a homeobox gene promotes growth of ovarian epithelial cells. Proc Natl Acad Sci U S A 2001; 98: 4060-5.173.	Espelund U, Cold S, Frystyk J, Orskov H, Flyvbjerg A. Elevated free IGF2 levels in localized, early-stage breast cancer in women. Eur J Endocrinol 2008; 159: 595-601.174.	Berteaux N, Aptel N, Cathala G, et al. A novel H19 antisense RNA overexpressed in breast cancer contributes to paternal IGF2 expression. Mol Cell Biol 2008; 28: 6731-45.175.	Ma XJ, Hilsenbeck SG, Wang W, et al. The HOXB13:IL17BR expression index is a prognostic factor in early-stage breast cancer. J Clin Oncol 2006; 24: 4611-9.176.	Chu P, Wu E, Weiss LM. Cytokeratin 7 and cytokeratin 20 expression in epithelial neoplasms: a survey of 435 cases. Mod Pathol 2000; 13: 962-72.177.	Sack MJ, Roberts SA. Cytokeratins 20 and 7 in the differential diagnosis of metastatic carcinoma in cytologic specimens. Diagn Cytopathol 1997; 16: 132-6.178.	Tot T. Cytokeratins 20 and 7 as biomarkers: usefulness in discriminating primary from metastatic adenocarcinoma. Eur J Cancer 2002; 38: 758-63.179.	Masuda TA, Kataoka A, Ohno S, et al. Detection of occult cancer cells in peripheral blood and bone marrow by quantitative RT-PCR assay for cytokeratin-7 in breast cancer patients. Int J Oncol 2005; 26: 721-30.180.	Moniaux N, Chakraborty S, Yalniz M, et al. Early diagnosis of pancreatic cancer: neutrophil gelatinase-associated lipocalin as a marker of pancreatic intraepithelial neoplasia. Br J Cancer 2008; 98: 1540-7.181.	Tong Z, Wu X, Ovcharenko D, Zhu J, Chen CS, Kehrer JP. Neutrophil gelatinase-associated lipocalin as a survival factor. Biochem J 2005; 391: 441-8.182.	Jung SS, Park HS, Lee IJ, et al. The HCCR oncoprotein as a biomarker for human breast cancer. Clin Cancer Res 2005; 11: 7700-8.183.	Chaib H, Cockrell EK, Rubin MA, Macoska JA. Profiling and verification of gene expression patterns in normal and malignant human prostate tissues by cDNA microarray analysis. Neoplasia 2001; 3: 43-52.184.	Calaf GM, Roy D. Human drug metabolism genes in parathion-and estrogen-treated breast cells. Int J Mol Med 2007; 20: 875-81.185.	Xu X, Wang B, Ye C, et al. Overexpression of macrophage migration inhibitory factor induces angiogenesis in human breast cancer. Cancer Lett 2008; 261: 147-57.186.	Iyengar P, Combs TP, Shah SJ, et al. Adipocyte-secreted factors synergistically promote mammary tumorigenesis through induction of anti-apoptotic transcriptional programs and proto-oncogene stabilization. Oncogene 2003; 22: 6408-23.187.	Asano T, Tada M, Cheng S, et al. Prognostic values of matrix metalloproteinase family expression in human colorectal carcinoma. J Surg Res 2008; 146: 32-42.188.	Decock J, Long JR, Laxton RC, et al. Association of matrix metalloproteinase-8 gene variation with breast cancer prognosis. Cancer Res 2007; 67: 10214-21.189.	Ye H, Yu T, Temam S, et al. Transcriptomic dissection of tongue squamous cell carcinoma. BMC Genomics 2008; 9: 69.190.	Martinez C, Bhattacharya S, Freeman T, Churchman M, Ilyas M. Expression profiling of murine intestinal adenomas reveals early deregulation of multiple matrix metalloproteinase (Mmp) genes. J Pathol 2005; 206: 100-10.191.	Schuetz CS, Bonin M, Clare SE, et al. Progression-specific genes identified by expression profiling of matched ductal carcinomas in situ and invasive breast tumors, combining laser capture microdissection and oligonucleotide microarray analysis. Cancer Res 2006; 66: 5278-86.192.	Yang YH, Deng H, Li WM, et al. Identification of matrix metalloproteinase 11 as a predictive tumor marker in serum based on gene expression profiling. Clin Cancer Res 2008; 14: 74-81.193.	Vazquez-Ortiz G, Pina-Sanchez P, Vazquez K, et al. Overexpression of cathepsin F, matrix metalloproteinases 11 and 12 in cervical cancer. BMC Cancer 2005; 5: 68.194.	Rizki A, Weaver VM, Lee SY, et al. A human breast cell model of preinvasive to invasive transition. Cancer Res 2008; 68: 1378-87.195.	Haupt LM, Irving RE, Weinstein SR, Irving MG, Griffiths LR. Matrix metalloproteinase localisation by in situ-RT-PCR in archival human breast biopsy material. Mol Cell Probes 2008; 22: 83-9.196.	Polette M, Gilles C, Nawrocki-Raby B, et al. Membrane-type 1 matrix metalloproteinase expression is regulated by zonula occludens-1 in human breast cancer cells. Cancer Res 2005; 65: 7691-8.197.	Tetu B, Brisson J, Wang CS, et al. The influence of MMP-14, TIMP-2 and MMP-2 expression on breast cancer prognosis. Breast Cancer Res 2006; 8: R28.198.	Szabova L, Yamada SS, Birkedal-Hansen H, Holmbeck K. Expression pattern of four membrane-type matrix metalloproteinases in the normal and diseased mouse mammary gland. J Cell Physiol 2005; 205: 123-32.199.	Sun YN, Li Y. Expression of mRNA for membrane-type 1, 2, and 3 matrix metalloproteinases in human laryngeal cancer. Chin Med Sci J 2004; 19: 170-3.200.	Jung M, Romer A, Keyszer G, et al. mRNA expression of the five membrane-type matrix metalloproteinases MT1-MT5 in human prostatic cell lines and their down-regulation in human malignant prostatic tissue. Prostate 2003; 55: 89-98.201.	Decock J, Hendrickx W, Drijkoningen M, et al. Matrix metalloproteinase expression patterns in luminal A type breast carcinomas. Dis Markers 2007; 23: 189-96.202.	Mendes O, Kim HT, Lungu G, Stoica G. MMP2 role in breast cancer brain metastasis development and its regulation by TIMP2 and ERK1/2. Clin Exp Metastasis 2007; 24: 341-51.203.	Jones JL, Glynn P, Walker RA. Expression of MMP-2 and MMP-9, their inhibitors, and the activator MT1-MMP in primary breast carcinomas. J Pathol 1999; 189: 161-8.204.	Bramhall SR, Neoptolemos JP, Stamp GW, Lemoine NR. Imbalance of expression of matrix metalloproteinases (MMPs) and tissue inhibitors of the matrix metalloproteinases (TIMPs) in human pancreatic carcinoma. J Pathol 1997; 182: 347-55.205.	Vaananen A, Srinivas R, Parikka M, et al. Expression and regulation of MMP-20 in human tongue carcinoma cells. J Dent Res 2001; 80: 1884-9.206.	Tjaderhane L, Palosaari H, Wahlgren J, Larmas M, Sorsa T, Salo T. Human odontoblast culture method: the expression of collagen and matrix metalloproteinases (MMPs). Adv Dent Res 2001; 15: 55-8.207.	Mendes O, Kim HT, Stoica G. Expression of MMP2, MMP9 and MMP3 in breast cancer brain metastasis in a rat model. Clin Exp Metastasis 2005; 22: 237-46.208.	Miyata Y, Iwata T, Ohba K, Kanda S, Nishikido M, Kanetake H. Expression of matrix metalloproteinase-7 on cancer cells and tissue endothelial cells in renal cell carcinoma: prognostic implications and clinical significance for invasion and metastasis. Clin Cancer Res 2006; 12: 6998-7003.209.	Gershtein ES, Korotkova EA, Shcherbakov AM, Prorokov VV, Golovkov DA, Kushlinskii NE. Matrix metalloproteinases 7 and 9 and their types 1 and 4 tissue inhibitors in tumors and plasma of patients with colorectal cancer. Bull Exp Biol Med 2007; 143: 459-62.210.	Shen D, He J, Chang HR. In silico identification of breast cancer genes by combined multiple high throughput analyses. Int J Mol Med 2005; 15: 205-12.211.	Simi L, Andreani M, Davini F, et al. Simultaneous measurement of MMP9 and TIMP1 mRNA in human non small cell lung cancers by multiplex real time RT-PCR. Lung Cancer 2004; 45: 171-9.212.	Hucl T, Brody JR, Gallmeier E, Iacobuzio-Donahue CA, Farrance IK, Kern SE. High cancer-specific expression of mesothelin (MSLN) is attributable to an upstream enhancer containing a transcription enhancer factor dependent MCAT motif. Cancer Res 2007; 67: 9055-65.213.	Dainty LA, Risinger JI, Morrison C, et al. Overexpression of folate binding protein and mesothelin are associated with uterine serous carcinoma. Gynecol Oncol 2007; 105: 563-70.214.	Ho M, Bera TK, Willingham MC, et al. Mesothelin expression in human lung cancer. Clin Cancer Res 2007; 13: 1571-5.215.	Diaz LK, Wiley EL, Morrow M. Expression of epithelial mucins Muc1, Muc2, and Muc3 in ductal carcinoma in situ of the breast. Breast J 2001; 7: 40-5.216.	Zhong XY, Kaul S, Bastert G. Evaluation of MUC1 and EGP40 in bone marrow and peripheral blood as a marker for occult breast cancer. Arch Gynecol Obstet 2001; 264: 177-81.217.	Schmid BC, Rudas M, Fabjani G, et al. Evaluation of MUC1 splice variants as prognostic markers in patients with ductal carcinoma in situ of the breast. Oncol Rep 2003; 10: 1981-5.218.	Li H, Cherukuri P, Li N, et al. Nestin is expressed in the basal/myoepithelial layer of the mammary gland and is a selective marker of basal epithelial breast tumors. Cancer Res 2007; 67: 501-10.219.	Biewenga P, Buist MR, Moerland PD, et al. Gene expression in early stage cervical cancer. Gynecol Oncol 2008; 108: 520-6.220.	Wang X, Li N, Li H, et al. Silencing of human phosphatidylethanolamine-binding protein 4 sensitizes breast cancer cells to tumor necrosis factor-alpha-induced apoptosis and cell growth arrest. Clin Cancer Res 2005; 11: 7545-53.221.	Li P, Wang X, Li N, et al. Anti-apoptotic hPEBP4 silencing promotes TRAIL-induced apoptosis of human ovarian cancer cells by activating ERK and JNK pathways. Int J Mol Med 2006; 18: 505-10.222.	Li H, Wang X, Li N, Qiu J, Zhang Y, Cao X. hPEBP4 resists TRAIL-induced apoptosis of human prostate cancer cells by activating Akt and deactivating ERK1/2 pathways. J Biol Chem 2007; 282: 4943-50.223.	Del Casar JM, Martin A, Garcia C, et al. Characterization of breast cancer subtypes by quantitative assessment of biological parameters: Relationship with clinicopathological characteristics, biological features and prognosis. Eur J Obstet Gynecol Reprod Biol 2008; 141: 147-52.224.	Hofling M, Lofgren L, von Schoultz E, Carlstrom K, Soderqvist G. Associations between serum testosterone levels, cell proliferation and progesterone receptor content in normal and malignant breast tissue in postmenopausal women. Gynecol Endocrinol 2008; 24: 405-10.225.	Wu G, Xing M, Mambo E, et al. Somatic mutation and gain of copy number of PIK3CA in human breast cancer. Breast Cancer Res 2005; 7: R609-16.226.	Adelaide J, Finetti P, Bekhouche I, et al. Integrated profiling of basal and luminal breast cancers. Cancer Res 2007; 67: 11565-75.227.	Moritani S, Ichihara S, Hasegawa M, et al. Serous papillary adenocarcinoma of the female genital organs and invasive micropapillary carcinoma of the breast. Are WT1, CA125, and GCDFP-15 useful in differential diagnosis? Hum Pathol 2008; 39: 666-71.228.	Caputo E, Manco G, Mandrich L, Guardiola J. A novel aspartyl proteinase from apocrine epithelia and breast tumors. J Biol Chem 2000; 275: 7935-41.229.	Lee BH, Hecht JL, Pinkus JL, Pinkus GS. WT1, estrogen receptor, and progesterone receptor as markers for breast or ovarian primary sites in metastatic adenocarcinoma to body fluids. Am J Clin Pathol 2002; 117: 745-50.230.	Clark JW, Snell L, Shiu RP, et al. The potential role for prolactin-inducible protein (PIP) as a marker of human breast cancer micrometastasis. Br J Cancer 1999; 81: 1002-8.231.	Nielsen BS, Rank F, Illemann M, Lund LR, Dano K. Stromal cells associated with early invasive foci in human mammary ductal carcinoma in situ coexpress urokinase and urokinase receptor. Int J Cancer 2007; 120: 2086-95.232.	Giannopoulou I, Mylona E, Kapranou A, et al. The prognostic value of the topographic distribution of uPAR expression in invasive breast carcinomas. Cancer Lett 2007; 246: 262-7.233.	Bhatavdekar JM, Patel DD, Shah NG, et al. Prolactin as a local growth promoter in patients with breast cancer: GCRI experience. Eur J Surg Oncol 2000; 26: 540-7.234.	Wood LD, Parsons DW, Jones S, et al. The genomic landscapes of human breast and colorectal cancers. Science 2007; 318: 1108-13.235.	Julien SG, Dube N, Read M, et al. Protein tyrosine phosphatase 1B deficiency or inhibition delays ErbB2-induced mammary tumorigenesis and protects from lung metastasis. Nat Genet 2007; 39: 338-46.236.	Fabre-Lafay S, Monville F, Garrido-Urbani S, et al. Nectin-4 is a new histological and serological tumor associated marker for breast cancer. BMC Cancer 2007; 7: 73.237.	Paruchuri V, Prasad A, McHugh K, Bhat HK, Polyak K, Ganju RK. S100A7-downregulation inhibits epidermal growth factor-induced signaling in breast cancer cells and blocks osteoclast formation. PLoS ONE 2008; 3: e1741.238.	Zafrakas M, Petschke B, Donner A, et al. Expression analysis of mammaglobin A (SCGB2A2) and lipophilin B (SCGB1D2) in more than 300 human tumors and matching normal tissues reveals their co-expression in gynecologic malignancies. BMC Cancer 2006; 6: 88.239.	Carter D, Douglass JF, Cornellison CD, et al. Purification and characterization of the mammaglobin/lipophilin B complex, a promising diagnostic marker for breast cancer. Biochemistry 2002; 41: 6714-22.240.	Baba F, Swartz K, van Buren R, et al. Syndecan-1 and syndecan-4 are overexpressed in an estrogen receptor-negative, highly proliferative breast carcinoma subtype. Breast Cancer Res Treat 2006; 98: 91-8.241.	Lo PK, Mehrotra J, D'Costa A, et al. Epigenetic suppression of secreted frizzled related protein 1 (SFRP1) expression in human breast cancer. Cancer Biol Ther 2006; 5: 281-6.242.	Kennedy S, Clynes M, Doolan P, et al. SNIP/p140Cap mRNA expression is an unfavourable prognostic factor in breast cancer and is not expressed in normal breast tissue. Br J Cancer 2008; 98: 1641-5.243.	Lien HC, Hsiao YH, Lin YS, et al. Molecular signatures of metaplastic carcinoma of the breast by large-scale transcriptional profiling: identification of genes potentially related to epithelial-mesenchymal transition. Oncogene 2007; 26: 7859-71.244.	Bergamaschi A, Tagliabue E, Sorlie T, et al. Extracellular matrix signature identifies breast cancer subgroups with different clinical outcome. J Pathol 2008; 214: 357-67.245.	Mirza M, Shaughnessy E, Hurley JK, et al. Osteopontin-c is a selective marker of breast cancer. Int J Cancer 2008; 122: 889-97.246.	Reinholz MM, Iturria SJ, Ingle JN, Roche PC. Differential gene expression of TGF-beta family members and osteopontin in breast tumor tissue: analysis by real-time quantitative PCR. Breast Cancer Res Treat 2002; 74: 255-69.247.	Streit M, Riccardi L, Velasco P, et al. Thrombospondin-2: a potent endogenous inhibitor of tumor growth and angiogenesis. Proc Natl Acad Sci U S A 1999; 96: 14888-93.248.	Bertin N, Clezardin P, Kubiak R, Frappart L. Thrombospondin-1 and -2 messenger RNA expression in normal, benign, and neoplastic human breast tissues: correlation with prognostic factors, tumor angiogenesis, and fibroblastic desmoplasia. Cancer Res 1997; 57: 396-9.249.	de Fraipont F, Nicholson AC, Feige JJ, Van Meir EG. Thrombospondins and tumor angiogenesis. Trends Mol Med 2001; 7: 401-7.250.	Ree AH, Florenes VA, Berg JP, Maelandsmo GM, Nesland JM, Fodstad O. High levels of messenger RNAs for tissue inhibitors of metalloproteinases (TIMP-1 and TIMP-2) in primary breast carcinomas are associated with development of distant metastases. Clin Cancer Res 1997; 3: 1623-8.251.	Inoue H, Mimori K, Shiraishi T, et al. Expression of tissue inhibitor of matrix metalloproteinase-1 in human breast carcinoma. Oncol Rep 2000; 7: 871-4.252.	Stevens TA, Meech R. BARX2 and estrogen receptor-alpha (ESR1) coordinately regulate the production of alternatively spliced ESR1 isoforms and control breast cancer cell growth and invasion. Oncogene 2006; 25: 5426-35.253.	Smid-Koopman E, Blok LJ, Chadha-Ajwani S, Helmerhorst TJ, Brinkmann AO, Huikeshoven FJ. Gene expression profiles of human endometrial cancer samples using a cDNA-expression array technique: assessment of an analysis method. Br J Cancer 2000; 83: 246-51.254.	Riddick AC, Shukla CJ, Pennington CJ, et al. Identification of degradome components associated with prostate cancer progression by expression analysis of human prostatic tissues. Br J Cancer 2005; 92: 2171-80.255.	Levy P, Vidaud D, Leroy K, et al. Molecular profiling of malignant peripheral nerve sheath tumors associated with neurofibromatosis type 1, based on large-scale real-time RT-PCR. Mol Cancer 2004; 3: 20.256.	Schultz H, Kahler D, Branscheid D, Vollmer E, Zabel P, Goldmann T. TKTL1 is overexpressed in a large portion of non-small cell lung cancer specimens. Diagn Pathol 2008; 3: 35.257.	He Q, Fornander T, Johansson H, et al. Thymidine kinase 1 in serum predicts increased risk of distant or loco-regional recurrence following surgery in patients with early breast cancer. Anticancer Res 2006; 26: 4753-9.258.	Chen CC, Chang TW, Chen FM, et al. Combination of multiple mRNA markers (PTTG1, Survivin, UbcH10 and TK1) in the diagnosis of Taiwanese patients with breast cancer by membrane array. Oncology 2006; 70: 438-46.259.	Seitz S, Wassmuth P, Fischer J, et al. Mutation analysis and mRNA expression of trail-receptors in human breast cancer. Int J Cancer 2002; 102: 117-28.260.	Ouellet V, Le Page C, Madore J, et al. An apoptotic molecular network identified by microarray: on the TRAIL to new insights in epithelial ovarian cancer. Cancer 2007; 110: 297-308.261.	Degen M, Brellier F, Kain R, et al. Tenascin-W is a novel marker for activated tumor stroma in low-grade human breast cancer and influences cell behavior. Cancer Res 2007; 67: 9169-79.262.	Degen M, Brellier F, Schenk S, et al. Tenascin-W, a new marker of cancer stroma, is elevated in sera of colon and breast cancer patients. Int J Cancer 2008; 122: 2454-61.263.	Rody A, Karn T, Ruckhaberle E, et al. Gene expression of topoisomerase II alpha (TOP2A) by microarray analysis is highly prognostic in estrogen receptor (ER) positive breast cancer. Breast Cancer Res Treat 2008.264.	Walker G, MacLeod K, Williams AR, Cameron DA, Smyth JF, Langdon SP. Estrogen-regulated gene expression predicts response to endocrine therapy in patients with ovarian cancer. Gynecol Oncol 2007; 106: 461-8.265.	Wei X, Xu H, Kufe D. Human mucin 1 oncoprotein represses transcription of the p53 tumor suppressor gene. Cancer Res 2007; 67: 1853-8.266.	Kourea HP, Koutras AK, Zolota V, et al. Expression of p27KIP1, p21WAF1 and p53 does not correlate with prognosis in node-negative invasive ductal carcinoma of the breast. Anticancer Res 2006; 26: 1657-68.267.	Silvestrini R, Benini E, Veneroni S, et al. p53 and bcl-2 expression correlates with clinical outcome in a series of node-positive breast cancer patients. J Clin Oncol 1996; 14: 1604-10.268.	Radvanyi L, Singh-Sandhu D, Gallichan S, et al. The gene associated with trichorhinophalangeal syndrome in humans is overexpressed in breast cancer. Proc Natl Acad Sci U S A 2005; 102: 11005-10.269.	Chang GT, Jhamai M, van Weerden WM, Jenster G, Brinkmann AO. The TRPS1 transcription factor: androgenic regulation in prostate cancer and high expression in breast cancer. Endocr Relat Cancer 2004; 11: 815-22.270.	Savinainen KJ, Linja MJ, Saramaki OR, et al. Expression and copy number analysis of TRPS1, EIF3S3 and MYC genes in breast and prostate cancer. Br J Cancer 2004; 90: 1041-6.271.	Roy R, Wewer UM, Zurakowksi D, Pories SE, Moses MA. ADAM 12 cleaves extracellular matrix proteins and correlates with cancer status and stage. J Biol Chem 2004; 279: 51323-30.272.	Bieche I, Lerebours F, Tozlu S, Espie M, Marty M, Lidereau R. Molecular profiling of inflammatory breast cancer: identification of a poor-prognosis gene expression signature. Clin Cancer Res 2004; 10: 6789-95.273.	Garvin S, Dabrosin C. In vivo measurement of tumor estradiol and vascular endothelial growth factor in breast cancer patients. BMC Cancer 2008; 8: 73.274.	Tringler B, Zhuo S, Pilkington G, et al. B7-h4 is highly expressed in ductal and lobular breast cancer. Clin Cancer Res 2005; 11: 1842-8.275.	Salceda S, Tang T, Kmet M, et al. The immunomodulatory protein B7-H4 is overexpressed in breast and ovarian cancers and promotes epithelial cell transformation. Exp Cell Res 2005; 306: 128-41.276.	Tringler B, Liu W, Corral L, et al. B7-H4 overexpression in ovarian tumors. Gynecol Oncol 2006; 100: 44-52.277.	Mugler KC, Singh M, Tringler B, et al. B7-h4 expression in a range of breast pathology: correlation with tumor T-cell infiltration. Appl Immunohistochem Mol Morphol 2007; 15: 363-70.278.	Galgano MT, Hampton GM, Frierson HF, Jr. Comprehensive analysis of HE4 expression in normal and malignant human tissues. Mod Pathol 2006; 19: 847-53.279.	Silberstein GB, Van Horn K, Strickland P, Roberts CT, Jr., Daniel CW. Altered expression of the WT1 wilms tumor suppressor gene in human breast cancer. Proc Natl Acad Sci U S A 1997; 94: 8132-7.280.	Miyoshi Y, Ando A, Egawa C, et al. High expression of Wilms' tumor suppressor gene predicts poor prognosis in breast cancer patients. Clin Cancer Res 2002; 8: 1167-71.281.	Dumur CI, Dechsukhum C, Wilkinson DS, Garrett CT, Ware JL, Ferreira-Gonzalez A. Analytical validation of a real-time reverse transcription-polymerase chain reaction quantitation of different transcripts of the Wilms' tumor suppressor gene (WT1). Anal Biochem 2002; 309: 127-36.282.	Lacroix M, Leclercq G. About GATA3, HNF3A, and XBP1, three genes co-expressed with the oestrogen receptor-alpha gene (ESR1) in breast cancer. Mol Cell Endocrinol 2004; 219: 1-7.283.	Doane AS, Danso M, Lal P, et al. An estrogen receptor-negative breast cancer subset characterized by a hormonally regulated transcriptional program and response to androgen. Oncogene 2006; 25: 3994-4008.284.	Frasor J, Chang EC, Komm B, et al. Gene expression preferentially regulated by tamoxifen in breast cancer cells and correlations with clinical outcome. Cancer Res 2006; 66: 7334-40.285.	Kornelia Polyak, personal communication.286.	Nathalie Scholler, personal communication.287.	Patrick Brown, personal communication.288.	Pavel Sova and André Lieber (ovarian stem cell signature), personal communication.289.	Samir Hanash and Martin McIntosh, personal communication.290.	CancerGene database (http://caroll.vjf.cnrs.fr/cancergene, this link is no longer functional).
