# Supplementary material for: Comparison of Breast Cancer to Healthy Control Tissue Discovers Novel Markers with Potential for Prognosis and Early Detection
Source: PLoS One. 2010 Feb 9;5(2):e9122. doi: 10.1371/journal.pone.0009122 (PMC2817747; doi:10.1371/journal.pone.0009122)
Supplement: Table S1 — Characteristics of the 64 patients who donated tissue. Age at diagnosis, Diagnosis (IDC = invasive ductal carcinoma, ILC = invasive lobular carcinoma, MET = metaplastic carcinoma, INF = inflammatory carcinoma, MUC = mucinous carcinoma, NML = normal), Stage, grade and tumor size noted during gross histopathological examination, Number of positive lymph nodes out of all tested, BI-RADS mammography score (1 = normal, 2 = benign, 3 = probably benign, 4 = suspicious, 5 = malignant), Breast Density score (0 = fatty, 1 = average, 2 = dense, 3 = very dense), Race (White, Black, Hispanic, Asian), Hormone receptor (estrogen and/or progesterone) and ERBB2 status, and the tissue(s) used (NI = normal ipsilateral, CA = invasive cancer, NC = normal contralateral, NM = normal from mammaplasty; fatty = 20-50% fat). Progression-free survival (PFI) and survival given in months. NED = no evidence of disease. Empty cells: no information. It should be noted that the breast reduction population was 15 years younger than the breast cancer population. (0.47 MB PDF) [file pone.0009122.s004.pdf]

Pt	Age	Diag-nosis	Stage	Grade	Tumor Size (cm)	LN pos.	BI-RADS	Breast Density	Race	HR Status	ERBB2 Status	Previous Cancer	Neo-adjuvant chemo	Chemo	Radia-tion	Recurrence	PFI	Survival since diagnosis	Vital Status	Tissue(s) per patient	NML in cancer cluster	Cancer in basal-enriched cluster	Cancer in luminal-like cluster
1	49	IDC	2a	3	1.7	1/25	5	1	W	+	-	Ovarian 32 mo prior		Yes	No	No		37	Alive NED	NI			
2	37	IDC	2a	2	0.8	1/14	5	2		+	+			Yes	No	No		50	Alive, Unknown Status	NI (fatty)			
3	44	IDC	2b	3	2.0	2/18	5	2	W	+	-	Cervical 25 mo prior		Yes	Yes	No		55	Alive NED	CA			*
4	81	IDC	2a	2	4.5	0/14	4	2	W	+	-			No	No	No		55	Alive NED	NI			
5	62	IDC	2b	3	4.0	2/11	4	2	W	-	+	Uterine 175 mo prior		No	No	Local	11	21	Expired w/ Breast CA	CA, NI (fatty)		*	
6	47	IDC	2c	3	7.0	19/36	4	0	W	-	-			Yes	Yes	Distant		21	Expired w/ Breast CA	CA		*	
7	37	IDC		3	5.5	6/10	4		W	-	+		*	Yes	No	Distant	7	28	Expired w/ Breast CA	NI	*		
8	56	IDC	2b	3	3.2	1/4	5	3	W	-	-			Yes	No	No		52	Alive NED	CA, NI		*	
9	60	IDLC	2b	3	2.2	1/24	2	3	W	+	-			Yes	No	No		52	Alive NED	CA			*
10	82	IDC	2b	3	5.0	3/18	5	2	W	-	-			Yes	Yes	No		52	Alive NED	CA		*	
11	52	DCIS		3	1.6	0/2	3	2	W	+				No	No	No		52	Alive NED	NI			
12	58	MET	2a	3	7.0	1/27	4	2	W	-	+			No	No	No		52	Alive NED	CA, NI		*	
13	36	IDC	2b	3	2.1, 1.5, 0.2	1/25	5	3	W	+	+			Yes	Yes	No		50	Alive NED	CA, NI	*		*
14	39	IDC	2a	3	0.8 - 5.1	2/19	5	1	W	+	-			Yes	No	Distant		52	Alive w/ Breast CA	CA, NI		*	
15	56	ILC	3c	2	10.0	16/28	4	2	W	+	-			Yes	Yes	Distant		52	Alive w/ Breast CA	NI (fatty)			
16	50	IDC	3c	3	Multifocal	15/19	4	3	W	+	-	Breast 1 mo prior	*	Yes	Yes	No		53	Alive NED	CA, NI, NC (fatty)	*		*
17	71	IDC	2b	3	4.7	2/35	5	2	B	+	-			No	No	Distant	9	12	Expired w/ Breast CA	CA, NI (fatty)		*	
18	60	ILC	2c	2	7.0	17/21	0	1	W	+	-			Yes	Yes	No		47	Alive NED	CA, NI			*
19	76	IDC	1	2	0.8	0/5	4	2	W	+	-	Skin 273 mo prior		No	No	No		47	Alive NED	NI (fatty)	*		
20	64	IDC	2b	3	3.7	1/17	4	2	W	-	+			Yes	Yes	No		47	Alive NED	CA, NI		*	
21	46	IDC			6.0	2/13	5	1	N	+	+			Yes	Yes	No		47	Alive NED	CA, NI (fatty)		*	
22	35	IDC	2b	2	3.8, 1.7	3/35	5	2	W	+	+			Yes	Yes	No		47	Alive NED	NI (fatty)			
23	71	IDC	1	2	2.0	0/3	3	2	W	+	-		*	Yes	No	No		52	Alive NED	NI			
24	79	MET	2a	3	7.5	1/10	5	1	B	-	-			Yes	No	Distant	3	5	Expired w/ Breast CA	CA, NI (fatty)		*	
25	48	IDC	1	2	1.1	0/21	1	2		+	+			Yes	No	No		45	Alive NED	NI			
26	56	IDLC	3	3	8.3		5		W	-	-		*	Yes	No	Local	7	10	Expired w/ Breast CA	CA, NI		*	
27	60	ILC	2a	2	4.5	0/8	5	2	W	+	-			Yes	Yes	Distant	7	42	Alive w/ Breast CA	CA, NI	*		*
28	67	INF	3b	2.5	3.0	5/18	5	1	W	+	-		*	Yes	Yes	No		48	Alive NED	NI			
29	57	IDC	1	3	2.0	0/3	5	2	W	-	-	Breast 64 mo prior		Yes	No	No		42	Alive NED	NI (fatty)	*		
30	55	IDC	2a	3	1.5	1/15	4	3	W	-	-		*	Yes	Yes	No		42	Alive NED	CA		*	
31	42	IDC	3a	3	4.7	7/14	0	1	W	+	+			Yes		No		41	Alive NED	NI, NI	*		
32	57	IDC	1	2.3	1.1	1/8	4	1	W	+	+	Breast 120 months prior		No	Yes	Never Disease Free	0		Expired w/ Breast CA	NI			
33	51	MUC	2b	1	6.0	0/3			W	+	-		*	Yes	No	No		43	Alive NED	CA, NI	*	*	
34	46	IDC	2a	3	1.8	2/25	4	2	W	-	+			Yes	No	No		41	Alive NED	NI (fatty)			
35	54	ILC	2	2	3.0	1/9	5	1	W	+	-			Yes	No	No		41	Alive NED	NI	*		
36	77	IDC	1	2.5	1.1	1/8	4	1	W	+	+	Breast 123 mo prior		No	Yes	Never Disease Free	0	140	Expired w/ Breast CA	NI			
37	65	IDC	1	3	2.0	0/2	4	1	W	+	+			No	Yes	No		38	Alive NED	NI, NI			
38	48	IDC	2a	3	1.7	1/5	4	3	W	+	-	Breast 1 mo prior		Yes	No	No		36	Alive NED	NC, NC-M	*		
39	64	IDC	2a	3	2.2	0/19	4	2	W	+	-	Skin, Vulva 66 mo prior		No	No	No		36	Alive NED	NI-A	*		
40	58	IDC	3a	3	4.2	4/29	5	2	W	-	-			Yes		No		35	Alive NED	CA, NI		*	
41	41	IDC	3c	3	5.2	13/31	3	2	W	-	+			Yes		No		34	Alive NED	CA		*	
42	32	IDC	3a	3	6.0	3/20	5	2	W	+	+			Yes	No	No		33	Alive NED	CA		*	
43	70	ILC	3c	2	5.7	24/28	5	2	W	+	-	Lymph node 123 mo prior		Yes		No		30	Alive NED	CA, CA, NI			*
44	48	DCIS	0	2		0/1			W	+				No	No	No		14	Alive NED	NI (fatty)			
45	61	NML							W											NM			
46		NML							W											NM			
47	45	NML																		NM			
48	32	NML																		NM			
49	43	NML																		NM			
50	46	NML																		NM			
51	28	NML																		NM			
52	55	NML							B											NM			
53	48	NML							B											NM			
54	20	NML							B											NM			
55	27	NML							A											NM			
56	57	NML							W											NM (fatty), NM (fatty)			
57		NML							W											NM			
58	52	NML																		NM, NM (fatty)			
59	35	NML																		NM, NM (fatty)			
60	27	NML							W											NM (fatty), NM (fatty)			
61	19	NML							W											NM (fatty), NM (fatty)			
62		NML							H											NM			
63		NML							B											NM, NM (fatty)			
64	44	NML							W											NM			

Table S1. *Characteristics of the 64 patients who donated tissue. Age at diagnosis, Diagnosis (IDC=invasive ductal carcinoma, ILC=invasive lobular carcinoma, MET=metaplastic carcinoma, INF=inflammatory carcinoma, MUC=mucinous carcinoma, NML=normal), Stage, grade and tumor size noted during gross histopathological examination, Number of positive lymph nodes out of all tested, BI-RADS mammography score (1=normal, 2=benign, 3= probably benign, 4= suspicious, 5=malignant), Breast Density score (0=fatty, 1=average, 2=dense, 3=very dense), Race (White, Black, Hispanic, Asian), Hormone receptor (estrogen and/or progesterone) and ERBB2 status, and the tissue(s) used (NI=normal ipsilateral, CA=invasive cancer, NC=normal contralateral, NM=normal from mammaplasty; fatty=20-50% fat). Progression-free survival (PFI) and survival given in months. NED=no evidence of disease. Empty cells: no information. It should be noted that the breast reduction population was 15 years younger than the breast cancer population.*
