# Supplementary material for: Comparison of Breast Cancer to Healthy Control Tissue Discovers Novel Markers with Potential for Prognosis and Early Detection
Source: PLoS One. 2010 Feb 9;5(2):e9122. doi: 10.1371/journal.pone.0009122 (PMC2817747; doi:10.1371/journal.pone.0009122)
Supplement: Table S2 — Tissue Characteristics. Histopathological examination was performed on the tissue piece adjacent to the one used for RNA extraction. Patient numbers in the first column match those in table S1. Tissue classification is explained in table S1. In the case of tumor tissue, the histology lists the percentage of the tissue specimen that was involved by tumor. The percentage of viable tumor cells, necrosis and the inflammatory component within the tumor histology is listed in the last three columns. These numbers donÊ¼t add up to 100% as we did not record the percentage of fibrous or fatty tissue. However, we limited collection of all tissues to those with less than 60% fat. Please note that tumor grade of an individual tissue piece might differ from the grade of the entire tumor listed in table S1. (0.31 MB PDF) [file pone.0009122.s005.pdf]

Pt	Tissue Classification	Histology	Grade	Of the prevailing cancer histology: % Viable Tumor Cells	% Necrosis	% Inflammatory Component
1	NI	100% normal				
2	NI (fatty)	100% normal fibroadipose, no glands				
3	CA	100% invasive ductal carcinoma	2	10%	10%	3%
4	NI	95% normal, 5% columnar cell change without atypia				
5	CA	100% invasive ductal carcinoma	3	60%	0%	3%
5	NI (fatty)	100% normal fibrofatty				0%
6	CA	60% invasive ductal carcinoma	3	15%	0%	10%
7	NI	100% fibroglandular tissue				
8	CA	100% invasive ductal carcinoma	3	70%	5%	5%
8	NI	100% normal				
9	CA	100% invasive lobular carcinoma	2	10%	0%	0%
10	CA	100% invasive ductal carcinoma	2	15%	0%	1%
11	NI	98% normal, 2% Proliferation without atypia				1%
12	CA	100% invasive ductal carcinoma	3	80%	20%	5%
12	NI	100% normal				
13	CA	80% invasive ductal carcinoma, 10% DCIS Cribriform, 10% normal	1	15%	0%	0%
13	NI	97% normal, 3% proliferation without atypia		0%	0%	5%
14	CA	100% invasive ductal carcinoma	3	25%	1%	1%
14	NI	100% normal				
15	NI (fatty)	100% adipose				
16	CA	90% invasive ductal carcinoma, 10% normal	2	15%	0%	2%
16	NI	100% fibroglandular				
16	NC (fatty)	100% adipose				
17	CA	90% invasive ductal carcinoma, 10% normal	3	70%	0%	5%
17	NI (fatty)	100% adipose				
18	CA	95% invasive mixed ductal/lobular carcinoma, 5% normal	2	15%	0%	2%
18	NI	100% fibroglandular				
19	NI (fatty)	100% adipose				
20	CA	80% invasive ductal carcinoma, 20% normal	2	30%	0%	5%
20	NI	100% normal				
21	CA	100% invasive ductal carcinoma	2	30%	0%	5%
21	NI (fatty)	100% normal				
22	NI (fatty)	100% fibroadipose				
23	NI	100% normal				
24	CA	95%% invasive metaplastic carcinoma, 5% normal	3	15%	0%	5%
24	NI (fatty)	100% fibroadipose				
25	NI	95% normal, 5% proliferation without atypia				
26	CA	100% invasive lobular carcinoma		95%	0%	0%
26	NI	100% normal				
27	CA	80% invasive lobular carcinoma, 20% normal	1	70%	0%	5%
27	NI	100% normal				
28	NI	100% normal				
29	NI (fatty)	100% adipose				
30	CA	100% invasive ductal carcinoma	2	15%	0%	1%
31	NI	100% normal				
31	NI	100% normal				
32	NI	100% normal				
33	CA	100% invasive mucinous carcinoma	1	5%	0%	0%
33	NI	100% normal				
34	NI (fatty)	100% adipose				
35	NI	100% normal				
36	NI	100% normal				
37	NI	100% breast parenchyma				
37	NI	100% breast parenchyma				
38	NC	95% normal, 5% proliferation without atypia				
38	NC-M	95% normal, 5% proliferation with atypia				
39	NI-A	95% normal, 5% proliferation with atypia				
40	CA	100% invasive ductal carcinoma	3	30%	0%	2%
40	NI	100% normal				
41	CA	100% invasive ductal carcinoma	3	80%	0%	5%
42	CA	90% invasive ductal carcinoma, 10% normal	2	15%	0%	
43	CA	100% invasive lobular carcinoma	2	7%	0%	15%
43	CA	100% invasive lobular carcinoma	2	15%	0%	2%
43	NI	95% normal, 5% proliferation without atypia				
44	NI (fatty)	100% fibroadenoma				5%
45	NM	100% normal				
46	NM	100% normal				
47	NM	100% normal				
48	NM	100% normal				
49	NM	100% normal				
50	NM	100% normal				
51	NM	100% normal				
52	NM	100% normal				
53	NM	100% normal				
54	NM	100% normal				
55	NM	100% normal				
56	NM (fatty)	100% fibroadipose				
56	NM (fatty)	100% fibroadipose				
57	NM	100% normal				
58	NM	100% normal				
58	NM (fatty)	100% fibroadipose				
59	NM	100% normal				
59	NM (fatty)	100% fibroadipose				
60	NM (fatty)	100% fibroadipose				
60	NM (fatty)	100% fibroadipose				
61	NM (fatty)	100% fibroadipose				
61	NM (fatty)	100% fibroadipose				
62	NM	100% normal				
63	NM	100% normal				
63	NM (fatty)	100% fibroadipose				
64	NM	100% normal				

Table S2. *Tissue Characteristics. Histopathological examination was performed on the tissue piece adjacent to the one used for RNA extraction. Patient numbers in the first column match those in table S1. Tissue classification is explained in table S1. In the case of tumor tissue, the histology lists the percentage of the tissue specimen that was involved by tumor. The percentage of viable tumor cells, necrosis and the inflammatory component within the tumor histology is listed in the last three columns. These numbers don't add up to 100% as we did not record the percentage of fibrous or fatty tissue. However, we limited collection of all tissues to those with less than 60% fat. Please note that tumor grade of an individual tissue piece might differ from the grade of the entire tumor listed in table S1.*
