# Supplementary material for: Comparison of Breast Cancer to Healthy Control Tissue Discovers Novel Markers with Potential for Prognosis and Early Detection
Source: PLoS One. 2010 Feb 9;5(2):e9122. doi: 10.1371/journal.pone.0009122 (PMC2817747; doi:10.1371/journal.pone.0009122)
Supplement: Table S3 — List of the 134 genes (by gene symbol) found by mining of expression data and/or LevelsDB mining (asterisk). References are listed in the Supplement. The 8 genes for which PCR was inconclusive are struck through. Thresholds for the cancers and controls were determined by the expression in the 28 normal mammaplasty tissues as mean +3 SD for genes with over-expression in the cancers and as below the minimum for genes with under-expression in the cancers. These cutoff values lie well above the gene expression variability in normal breast tissues listed in Table 1. Columns 2 and 3 list the percent of cancer or control tissues above or below this threshold. Result column: over- or under-expression in cancer tissue for genes with > = 20% of the cancers and < = 5% of the controls above or below threshold. (0.06 MB PDF) [file pone.0009122.s006.pdf]

Gene Symbol	Cancers	Con-trols	Re-sult	Gene Symbol	Can-cers	Con-trols	Re-sult	Gene Symbol	Can-cers	Con-trols	Result
ADAM12*	4%	0%		EPOR	36%	0%	∇	PEBP1	56%	0%	∇
AGR2*	16%	4%		~~ERBB2~~				PEBP4	0%	0%	
AKT1	0%	4%		ERBB3*	4%	0%		PGR	36%	0%	∇
AMBP*	0%	0%		ERBB4	44%	0%	∇	PIK3CA	12%	0%	
ANGPT2*	44%	0%	∇	ESR1	28%	4%	Δ	PIP	0%	0%	
APOL1*	24%	0%	∇	ETAA1*	52%	0%	∇	PLAUR	36%	0%	Δ
AR	48%	0%	∇	FGFR2*	20%	0%	∇	~~PRL~~			
ASPN*	36%	4%	Δ	FN1*	56%	0%	Δ	PRLR*	8%	0%	
BGN*	32%	4%	Δ	FOXA1	28%	0%	Δ	PROCR	88%	0%	∇
BIRC5	28%	4%	Δ	GATA3	32%	0%	Δ	PSMA5*	4%	0%	
~~BRCA1~~				GDF15	12%	4%		PTPN1*	8%	0%	
BRCA2	4%	4%		HOXB7	20%	0%	∇	PVRL4	0%	0%	
BRMS1	8%	0%		IFIT1*	4%	0%		S100A7	56%	8%	Δ
BUB1	72%	0%	Δ	IGF2	8%	0%		S100B*	60%	0%	∇
C18orf8*	4%	0%		~~KLK3~~				SCGB2A1	4%	4%	
CALB2	0%	0%		KRT20	0%	4%		SCUBE2*	52%	0%	∇
CAV1	84%	0%	∇	KRT7	0%	0%		SDC1	16%	0%	
CCNE1	28%	0%	Δ	LCN2	0%	0%		SFRP1	76%	0%	∇
CD274	24%	0%	Δ	LETMD1	84%	0%	∇	SFRP2*	4%	0%	
CD44	20%	0%	∇	LPAR3	4%	4%		SNIP	16%	8%	
CDH1	0%	0%		LRRC15*	68%	0%	Δ	SPARC	52%	0%	∇
CDKN1B*	28%	0%	∇	LTF*	4%	0%		SPP1	32%	0%	Δ
CDX2	0%	4%		LYZ*	12%	4%		STC2*	0%	0%	
CFB*	24%	0%	Δ	MGST1	48%	0%	∇	SUMF2*	0%	0%	
COL11A1*	60%	4%	Δ	MIF	8%	0%		THBS2	4%	0%	
COL1A1*	68%	0%	Δ	MMP1	56%	4%	Δ	TIMP1	24%	0%	Δ
COL1A2*	40%	4%	Δ	MMP10	32%	4%	Δ	TIMP2	48%	0%	∇
COL3A1*	0%	0%		MMP11	68%	4%	Δ	TIMP3	72%	0%	∇
COL5A1*	20%	0%	Δ	MMP12	56%	4%	Δ	TIMP4	68%	0%	∇
COL5A2*	28%	0%	∇	MMP13	60%	0%	Δ	TK1	36%	0%	Δ
COL6A3*	12%	0%		MMP14	4%	0%		TM9SF2*	16%	0%	
COL8A1*	16%	0%		MMP16	0%	4%		TNFRSF10B	20%	0%	∇
COMP*	64%	0%	Δ	~~MMP17~~				TNN	88%	0%	∇
CSNK2A1	16%	0%		MMP2	24%	0%	∇	TOP2A	52%	0%	Δ
CTGF	20%	0%	∇	MMP20	12%	0%		TP53	8%	0%	
CTHRC1*	48%	0%	Δ	MMP3	12%	4%		TRPS1	0%	0%	
~~CXCL1~~				MMP7	20%	4%	∇	TTF1	8%	4%	
CYP4B1*	40%	0%	∇	MMP8	8%	0%		VCAN*	40%	0%	Δ
CYR61	36%	0%	∇	MMP9	16%	0%		VEGFA	0%	0%	
~~DCD*~~				MSLN	16%	4%		VTCN1	0%	0%	
DEFA1	0%	0%		MUC1	44%	4%	Δ	WFDC2	20%	4%	Δ
DEFA3	0%	0%		~~MYBL2*~~				WT1	48%	4%	Δ
ECM1*	4%	4%		NES	36%	0%	∇	XBP1	4%	0%	
EGFR	72%	0%	∇	OAS1*	20%	0%		YWHAZ	12%	0%	
EPO	8%	0%		OAS2*	16%	0%	Δ				

***Table S3**: List of the 134 genes (by gene symbol) found by mining of expression data and/or LevelsDB mining (asterisk). References are listed in the Supplement. The 8 genes for which PCR was inconclusive are struck through. Thresholds for the cancers and controls were determined by the expression in the 28 normal mammaplasty tissues as mean +3 SD for genes with over-expression in the cancers and as below the minimum for genes with under-expression in the cancers. These cutoff values lie well above the gene expression variability in normal breast tissues listed in Table 1. Columns 2 and 3 list the percent of cancer or control tissues above or below this threshold. Result column: over- (Δ) or under- (∇) expression in cancer tissue for genes with ≥20% of the cancers and ≤5% of the controls above or below threshold.*
