# Supplementary material for: Comparison of Breast Cancer to Healthy Control Tissue Discovers Novel Markers with Potential for Prognosis and Early Detection
Source: PLoS One. 2010 Feb 9;5(2):e9122. doi: 10.1371/journal.pone.0009122 (PMC2817747; doi:10.1371/journal.pone.0009122)
Supplement: Table S4 — Comparison of PCR results from the original PCR and the OpenArray results from the reduced tissue and gene set. Listed are the expression values for the tumors (pink and orange shading in the leftmost column) and the mammaplasty normal controls (green shading) for both the original (TOR) and the OpenArray platform (BT). The yellow- and purple-shaded cells represent genes with over- and under-expression respectively. Due to the extreme low number of control tissues (9), values with high error (outliers) have a huge influence on the cutoff and hence the overall results. Four of these outliers were removed (empty cells). (0.25 MB PDF) [file pone.0009122.s007.pdf]

Tissue	AKT1		AR		BIRC5		BRCA1		BRCA2		BUB1		CAV1	
	TOR	BT	TOR	BT	TOR	BT	TOR	BT	TOR	BT	TOR	BT	TOR	BT
P03 T03	0.7	1.0	1.6	1.4	0.7	0.2	0.6	1.4	0.7	0.9	0.5	0.7	0.5	0.6
P05 T05	0.7	0.8	0.0	0.1	31.3	9.5	0.6	0.7	1.7	1.3	5.5	4.7	0.4	0.4
P09 T11	0.9	1.0	1.3	1.4	1.6	19.8	2.2	1.7	1.5	1.6	1.3	1.2	1.0	0.9
P13 T16	1.1	0.7	1.2	0.9	0.9	3.1	0.4	0.3	1.2	0.6	1.3	0.8	0.4	0.3
P14 T18	1.6	1.3	0.2	0.6	2.2	8.4	1.7	1.1	0.4	1.4	2.1	1.7	0.4	0.6
P16 T22	1.3	1.9	0.8	0.9	3.2	11.2	1.2	2.3	0.8	1.2	0.9	1.9	1.4	2.0
P17 T24	1.7	1.8	0.2	0.3	33.5	25.3	2.1	3.4	3.0	1.4	2.9	3.3	0.5	0.5
P20 T29	1.3	1.1	1.1	1.5	2.4	5.3	1.3	1.0	2.2	1.1	1.8	1.5	0.6	0.5
P21 T31	4.1	1.5	0.5	1.3	8.9	0.2	2.0	1.8	0.8	1.1	1.7	1.7	0.1	0.3
P27 T40	0.9	1.1	1.6	2.1	0.0	0.5	1.9	2.0	1.5	0.1	0.8	1.5	1.0	1.1
P30 T44	1.0	0.8	0.1	0.9	5.0	0.3	1.1	0.8	0.0	5.3	1.4	1.2	0.4	0.0
P41 T60	0.9	0.6	2.6	1.6	7.4	5.3	0.9	0.8	0.9	1.1	2.0	2.4	0.2	0.3
P43 T63	1.5	1.0	1.6	1.6	7.4	11.1	1.5	1.5	0.6	0.1	1.3	1.7	0.9	1.2
P49 T70	0.7	0.8	1.3	1.1	0.0	0.8	0.6	0.6	1.1	0.1	0.2	0.0	7.9	7.1
P52 T73	1.0	1.6	0.9	1.4	0.5	1.2	0.3	0.9	0.8	0.2	0.2	0.0	7.3	8.8
P54 T75	0.8	1.0	0.5	0.6	1.1	0.6	0.8	1.0	1.2	1.7	1.1	0.6	2.9	2.7
P56 T77	0.7	1.2	1.1	1.2	0.3	0.7	0.7	1.4	0.8	0.1	0.1	0.3	11.4	10.0
P59 T82	1.4	1.0	1.5	1.2	0.2	0.5	1.1	0.6	1.1	2.1	0.1	0.2	2.8	2.9
P60 T84	1.0	0.8	1.6	1.4	0.0	0.7	0.6	0.9	0.6	0.1	0.1	0.3	9.6	7.0
P62 T88	0.7	0.8	0.8	0.7	0.0		0.6	0.5	0.7	0.1	0.1	0.2	9.1	7.6
P63 T91	0.6	0.8	0.8	0.7	0.3	0.5	0.7	0.9	1.9	0.1	0.4	0.2	3.1	
P62 T89	1.4	0.9	0.3	0.8	0.3	0.5	0.7	0.9	0.7	1.9	0.4	0.3	3.9	4.0

Tissue	CCNE1		CD44		CDH1		CDKN1B		COL1A1		CYR61		EGFR	
	TOR	BT	TOR	BT	TOR	BT	TOR	BT	TOR	BT	TOR	BT	TOR	BT
P03 T03	0.7	0.6	1.0	1.2	1.3	2.0	1.2	1.3	3.7	1.9	0.4	0.8	0.3	0.4
P05 T05	9.9	5.8	0.8	0.8	1.0	1.4	0.8	0.9	0.0	0.5	0.3	0.4	1.4	0.9
P09 T11	1.0	0.7	1.3	1.1	1.3	1.4	1.3	1.7	0.0	1.6	1.0	0.8	0.8	0.9
P13 T16	0.8	0.5	0.4	0.3	1.5	1.6	1.4	0.9	0.0	1.7	0.6	0.4	0.4	0.3
P14 T18	0.8	0.8	0.6	0.7	1.8	1.9	0.6	0.8	10.6	2.7	1.7	1.6	0.3	0.3
P16 T22	1.7	1.1	0.7	0.5	0.9	1.1	1.1	1.2	0.0	0.7	0.6	0.7	0.5	0.9
P17 T24	4.4	3.1	0.3	0.2	1.0	1.0	1.1	0.9	0.0	0.5	0.1	0.1	0.3	0.2
P20 T29	1.0	1.9	0.6	0.8	1.3	1.7	0.5	0.7	0.0	1.1	1.2	1.1	0.9	0.7
P21 T31	2.0	1.2	0.7	0.5	1.3	0.8	1.1	1.0	2.4	0.5	0.4	0.5	0.2	0.1
P27 T40	0.7	0.9	1.1	1.2	0.7	0.7	0.6	0.7	3.9	3.0	1.2	1.2	1.0	1.1
P30 T44	2.5	1.9	1.9	1.6	0.9	0.5	0.7	0.5	0.0	0.0	0.8	0.8	1.7	2.4
P41 T60	2.8	2.4	0.7	0.8	1.5	1.9	0.7	0.8	0.0	0.6	0.5	0.6	0.7	0.8
P43 T63	0.7	0.6	1.4	1.1	0.4	0.4	1.8	1.7	0.0	1.4	0.8	1.1	0.8	0.4
P49 T70	0.4	0.9	1.4	1.2	0.2	0.1	0.9	0.8	0.0	0.5	1.9	1.7	1.6	1.3
P52 T73	0.6	1.3	0.6	0.8	0.0	0.0	0.6	1.0	0.0	0.4	0.9	0.8	1.6	3.2
P54 T75	1.0	0.6	1.2	1.0	1.5	1.0	1.3	1.0	0.0	1.6	4.9	4.2	1.4	2.5
P56 T77	1.5	1.6	0.7	0.9	0.3	0.2	1.0	1.3	0.0	0.3	3.3	2.3	2.1	3.6
P59 T82	0.5	0.8	1.0	1.0	1.6	2.7	1.0	1.4	0.0	0.4	1.0	1.3	1.0	1.8
P60 T84	0.5	0.6	1.1	1.5	0.0	0.2	1.0	1.2	0.0	0.7	2.0	2.8	1.7	2.5
P62 T88	0.6	1.2	1.0	1.3	0.3	0.4	0.7	1.0	0.0	0.5	3.7	4.7	2.7	4.0
P63 T91	1.1	0.5	1.1	1.0	1.3	1.0	1.3	1.2	0.0	0.3	3.1	3.0	2.4	2.1
P62 T89	0.7	1.1	1.5	1.1	1.0	0.8	1.1	1.3	0.0	0.2	3.9	3.4	2.3	3.8

Tissue	ERBB3		ERBB4		ESR1		FN1		GDF15		IGF2		LCN2	
	TOR	BT	TOR	BT	TOR	BT	TOR	BT	TOR	BT	TOR	BT	TOR	BT
P03 T03	1.6	1.3	0.5	0.7	5.7	9.4	3.6	4.1	2.3	4.2	0.5	0.3	0.7	1.2
P05 T05	1.2	0.8	0.0	0.0	0.1	0.0	0.8	0.9	0.9	0.2	0.5	0.1	10.8	12.7
P09 T11	1.2	2.2	3.5	4.3	3.6	7.4	1.3	0.8	0.0	1.8	2.2	1.3	0.0	1.5
P13 T16	1.6	1.0	2.4	1.7	2.4	2.1	4.5	2.4	2.7	2.6	0.6	0.4	1.0	0.8
P14 T18	0.6	1.0	0.4	0.6	1.1	5.9	6.2	4.8	0.0	0.6	1.4	0.2	0.5	1.0
P16 T22	0.8	1.1	4.9	4.2	4.1	6.1	0.9	1.1	0.0	0.9	0.9	0.9	1.6	1.3
P17 T24	1.1	1.0	1.0	1.1	7.5	12.3	3.1	1.0	1.0	1.4	0.8	0.2	0.0	0.0
P20 T29	0.4	0.6	0.5	0.9	0.2	0.2	1.9	1.4	0.0	1.4	0.8	0.2	0.3	0.7
P21 T31	2.6	2.1	0.9	0.6	1.0	2.9	1.2	1.2	1.4	0.5	0.5	0.1	1.9	2.2
P27 T40	1.0	2.2	3.9	5.7	3.4	6.3	2.2	2.2	0.0	0.7	1.4	1.1	2.0	1.0
P30 T44	0.5	0.8	0.3	0.1	0.1	0.3	1.2	3.2	0.0	3.1	2.3	0.3	10.6	0.0
P41 T60	1.0	0.9	0.0	0.0	0.1	0.1	3.3	3.5	1.4	0.1	0.3	0.1	2.0	2.0
P43 T63	1.7	2.2	1.2	2.2	4.3	4.9	0.7	1.2	8.3	0.8	1.0	1.0	1.0	0.8
P49 T70	0.1	0.3	0.7	0.6	0.5	0.6	0.6	0.3	0.0	0.3	2.3	1.0	0.8	1.2
P52 T73	0.0	0.0	1.0	1.5	0.2	1.1	0.4	0.5	0.0	0.3	2.4	2.5	0.1	0.0
P54 T75	1.3	1.3	2.5	1.9	0.4	0.5	0.5	0.5	0.0	0.3	1.1	1.1	1.0	0.8
P56 T77	0.1	0.3	0.6	0.5	0.6	0.9	0.2	0.3	0.0	0.3	1.2	1.4	0.4	0.0
P59 T82	0.6	1.2	2.2	3.9	2.8	2.0	0.3	0.5	0.0	0.4	0.8	2.0	0.9	0.9
P60 T84	0.0	0.1	0.5	0.6	1.0	0.6	1.0	1.0	0.0	0.3	0.9	1.0	0.2	0.7
P62 T88	0.2	0.5	1.1	0.8	0.6	0.6	0.8	0.7	0.0	0.2	1.2	1.3	1.0	0.8
P63 T91	1.2	1.0	2.9	2.1	1.1	0.9	1.0	0.3	0.0	0.3	1.2	1.1	8.9	9.3
P62 T89	0.5	1.0	1.3	1.2	0.8	0.7	0.3	0.4	0.0	0.6	1.0	1.5	1.7	1.6

Tissue	LTF		MGST1		MMP1		MMP11		MMP14		MMP17	
	TOR	BT	TOR	BT	TOR	BT	TOR	BT	TOR	BT	TOR	BT
P03 T03	1.0	1.5	0.2	0.2	3.6	12.1	0.8	4.6	0.5	3.4	0.7	1.2
P05 T05	0.5	0.4	1.0	1.2	2.7	4.7	0.6	0.5	0.4	0.4	0.4	0.4
P09 T11	0.8	0.9	1.0	0.5	0.6	1.2	0.0	1.3	1.4	1.5	0.0	0.1
P13 T16	0.3	0.2	0.2	0.3	0.6	0.6	1.2	1.6	1.3	2.0	1.3	1.1
P14 T18	0.1	0.1	0.1	0.1	2.9	7.9	2.7	7.8	1.7	6.5	1.2	2.4
P16 T22	1.9	1.9	1.6	1.1	1.7	1.1	0.3	1.6	0.8	1.8	0.9	1.2
P17 T24	0.0	0.0	0.7	0.8	1.7	2.4	1.2	2.0	0.6	1.8	0.7	0.6
P20 T29	0.3	0.3	1.0	1.3	13.6	44.4	0.9	2.2	0.8	2.4	0.0	0.9
P21 T31	3.2	3.2	1.0	1.0	4.5	9.5	2.6	1.2	0.6	0.4	0.7	1.5
P27 T40	2.5	3.0	0.3	0.4	0.0	0.0	0.3	1.7	3.6	3.5	2.4	1.4
P30 T44	0.9	0.7	0.5	0.0	15.7	27.6	4.4	2.7	1.5	2.5	0.9	0.0
P41 T60	1.2	1.0	1.0	1.4	1.7	5.8	1.2	2.6	0.3	1.7	0.0	0.0
P43 T63	1.1	1.0	1.0	1.0	1.4	1.0	0.0	0.8	0.4	0.0	1.1	0.7
P49 T70	0.6	1.1	3.6	2.6	0.0	0.1	0.0	0.2	1.8	0.0	0.0	1.8
P52 T73	0.1	0.4	2.9	2.6	0.0	0.1	0.0	0.2	2.9	0.0	0.0	0.1
P54 T75	5.3	4.7	1.2	0.7	0.4	1.0	0.0	0.1	1.5	0.0	0.0	0.1
P56 T77	3.6	4.5	3.4	3.3	0.2	0.1	0.0	0.1	1.0	0.0	3.6	0.1
P59 T82	1.8		1.3	1.4	0.2	0.0	0.0	0.1	0.5	0.8	2.6	0.1
P60 T84	0.1	0.2	2.8	3.5	0.5	0.1	0.0	0.1	1.0	1.2	2.6	1.9
P62 T88	1.0	3.6	2.6	2.8	0.0	0.1	0.0	0.1	0.6	0.3	0.0	1.8
P63 T91	5.9	6.0	0.7	0.3	0.0	0.0	0.0	0.1	0.6	1.6	3.3	1.5
P62 T89	3.2	6.3	1.9	1.2	0.0	0.0	0.0	0.0	1.5	0.8	0.0	1.5

Tissue	MMP2		MMP9		MUC1		MYBL2		PGR		PIK3CA		SCUBE2	
	TOR	BT	TOR	BT	TOR	BT	TOR	BT	TOR	BT	TOR	BT	TOR	BT
P03 T03	0.9	1.1	0.6	1.4	2.0	3.9	0.3	0.5	3.8	3.0	0.8	1.6	0.7	1.3
P05 T05	0.3	0.4	0.9	2.1	0.6	0.6	3.1	6.6	0.1	0.3	0.9	0.8	0.1	0.1
P09 T11	1.2	1.1	1.0	1.0	2.9	4.1	1.8	1.8	5.0	1.3	0.0	1.4	3.3	7.0
P13 T16	1.6	1.2	1.5	1.1	1.8	2.7	1.2	0.7	2.7	1.7	1.2	0.4	1.3	1.6
P14 T18	1.5	1.7	4.0	8.9	1.7	2.5	1.3	3.3	2.7	0.0	0.9	0.0	0.4	0.8
P16 T22	0.9	0.9	1.0	1.0	1.6	4.0	1.6	2.9	2.9	2.0	0.8	0.8	2.3	5.4
P17 T24	0.4	0.3	2.4	4.7	0.1	0.1	6.1	10.9	0.4	0.4	0.8	1.1	0.3	0.2
P20 T29	1.2	1.0	1.6	2.0	0.6	0.6	3.7	7.3	0.2	0.0	0.8	0.5	0.3	0.2
P21 T31	0.4	0.4	0.7	0.9	2.5	3.2	3.3	4.0	0.4	0.4	0.7	0.8	0.7	0.7
P27 T40	2.8	1.9	0.8	0.5	5.8	6.2	0.6	1.2	4.8	1.9	1.3	1.5	3.3	7.7
P30 T44	1.6	1.6	2.1	2.6	2.3	2.6	5.3	0.0	0.4	0.0	1.3	0.7	0.5	0.0
P41 T60	0.7	0.8	1.2	2.6	1.8	4.0	3.3	7.6	0.1	0.0	0.7	0.5	0.3	0.4
P43 T63	1.1	0.9	0.2	0.2	3.4	3.7	1.4	1.9	1.4	0.7	0.0	0.0	7.4	10.0
P49 T70	2.1	1.5	1.9	1.2	0.3	0.1	0.0	0.0	0.8	2.0	1.8	1.5	1.0	1.1
P52 T73	0.5	0.9	0.5	0.8	0.1	0.1	0.0	0.8	1.0	2.7	1.5	2.3	0.9	2.2
P54 T75	2.3	1.5	0.7	0.5	0.9	0.7	0.3	0.8	1.8	1.4	1.7	1.0	1.0	0.8
P56 T77	0.8	1.0	1.1	0.9	0.3	0.3	0.5	0.6	0.6	0.1	1.8	1.3	1.0	0.7
P59 T82	0.8	0.7	1.1	1.4	0.7	0.9	0.1	0.6	2.7	2.7	0.9	1.1	2.6	6.1
P60 T84	2.2	3.0	1.4	1.0	0.1	0.1	0.0	0.5	0.7	0.1	1.6		1.0	1.4
P62 T88	0.9	1.2	0.9	0.4	0.4	0.5	0.5	0.4	0.5	0.1	1.6	1.8	1.8	1.7
P63 T91	0.9	0.9	0.6	0.2	0.9	1.1	0.8	0.5	2.5	1.5	1.3	1.0	1.0	0.7
P62 T89	1.2	1.1	0.4	0.3	0.5	0.6	0.0	0.4	1.0	1.8	2.0	1.0	2.3	1.8

Tissue	TIMP1		TIMP3		TK1		TNFRSF10B		VEGFA		WT1		YWHAZ	
	TOR	BT	TOR	BT	TOR	BT	TOR	BT	TOR	BT	TOR	BT	TOR	BT
P03 T03	0.6	1.5	0.9	1.0	1.1	0.9	0.9	1.3	0.7	0.8	2.8	5.1	0.6	1.0
P05 T05	0.7	1.1	0.3	0.0	3.1	1.9	0.5	0.6	0.5	0.8	7.9	11.8	1.3	1.6
P09 T11	2.1	2.4	1.3	0.1	7.9	1.7	1.4	1.1	0.2	1.0	6.5	3.4	1.1	0.9
P13 T16	1.2	1.2	0.8	0.6	0.4	0.6	1.5	1.3	1.2	0.8	3.0	2.5	0.8	0.8
P14 T18	1.1	1.7	1.4	0.1	3.1	1.3	0.5	0.4	1.2	1.8	7.4	5.9	1.3	1.8
P16 T22	1.2	1.5	1.0	2.5	1.4	1.8	1.0	1.0	1.0	0.9	0.6	0.1	1.4	1.7
P17 T24	1.0	1.0	0.3	0.0	5.5	4.0	0.7	0.6	0.9	0.8	0.9	1.2	1.1	1.3
P20 T29	0.8	1.8	0.5	1.0	4.7	2.0	0.8	0.7	1.5	1.1	3.3	3.0	2.2	3.2
P21 T31	0.7	0.4	0.4	0.7	0.0	1.3	0.8	0.6	0.4	0.9	1.7	1.2	2.0	2.5
P27 T40	1.6	1.0	1.0	1.7	0.6	2.4	1.1	1.4	0.7	1.2	1.8	2.2	0.8	0.8
P30 T44	1.8	1.6	0.6	2.9	2.8	0.0	1.1	0.8	1.0	1.3	7.5	4.5	0.9	0.9
P41 T60	0.8	0.9	0.3	0.6	2.9	1.8	0.6	0.5	2.6	2.2	4.8	6.0	1.0	1.6
P43 T63	1.1	1.1	0.7	0.1	6.2	1.0	0.9	0.8	0.5	0.6	16.8	16.0	1.4	1.3
P49 T70	1.0	0.6	2.6	0.1	0.0	0.6	1.1	1.1	0.9	0.5	0.0	0.2	0.9	1.0
P52 T73	0.7	0.9	2.2	4.1	0.0	0.7	0.8	0.7	1.2	1.9	0.0	0.2	0.9	1.0
P54 T75	0.9	0.9	1.7	1.6	0.9	0.9	1.8	1.7	1.1	1.2	0.4	0.1	1.0	0.7
P56 T77	0.5	0.7	1.9	2.1	0.6	0.4	1.0	0.9	2.2	2.0	0.9	0.1	1.1	1.3
P59 T82	1.0	0.9	1.2	2.2	2.2	0.3	1.0	1.1	0.0	1.0	0.0	0.1	0.9	0.8
P60 T84	1.0	1.3	2.2	3.2	0.4	0.6	0.9	1.0	1.9	1.8	0.0	0.1	0.7	1.1
P62 T88	1.0	1.0	2.4	0.1	0.3	0.8	1.0	1.2	4.7	3.2	0.6	0.1	0.9	1.1
P63 T91	0.8	0.7	1.5	1.6	0.3	0.8	1.4	1.2	0.8	0.9	0.0	0.1	1.0	0.6
P62 T89	0.5	1.0	2.2	5.2	0.5	1.0	1.2	1.4	1.8	1.7	0.0	0.1	1.3	1.0

Table S4*: Comparison of PCR results from the original PCR and the OpenArray results from the reduced tissue and gene set. Listed are the expression values for the tumors (pink and orange shading in the left-most column) and the mammaplasty normal controls (green shading) for both the original (TOR) and the OpenArray platform (BT). The yellow- and purple-shaded cells represent genes with over- and under-expression respectively. Due to the extreme low number of control tissues (9), values with high error (outliers) have a huge influence on the cutoff and hence the overall results. Four of these outliers were removed (empty cells).*
