# Supplementary material for: Comparison of Breast Cancer to Healthy Control Tissue Discovers Novel Markers with Potential for Prognosis and Early Detection
Source: PLoS One. 2010 Feb 9;5(2):e9122. doi: 10.1371/journal.pone.0009122 (PMC2817747; doi:10.1371/journal.pone.0009122)
Supplement: Table S5 — Percent CV values of original and unfiltered OpenArray PCR results. Percent CV values for all tissues (%CV a) and for only those tissues with outliers were removed from the OpenArray results (%CV b). Removal was acceptable because the OpenArray PCR was not duplicated like the original PCR and had a higher number of values close or below the PCRÊ¼s detection limit. (0.04 MB PDF) [file pone.0009122.s008.pdf]

Symbol	%CV (a)	%CV (b)		Symbol	%CV (a)	%CV (b)
**AKT1**	21%	21%		**LTF**	36%	31%
**AR**	32%	32%		**MGST1**	26%	21%
**BIRC5**	74%	60%		**MMP1**	80%	60%
**BRCA1**	23%	23%		**MMP11**	97%	65%
**BRCA2**	71%	67%		**MMP14**	73%	71%
**BUB1**	42%	42%		**MMP17**	82%	52%
**CAV1**	29%	19%		**MMP2**	14%	14%
**CCNE1**	32%	32%		**MMP9**	36%	36%
**CD44**	20%	20%		**MUC1**	25%	25%
**CDH1**	26%	26%		**MYBL2**	65%	45%
**CDKN1B**	19%	19%		**PGR**	69%	69%
**COL1A1**	125%	64%		**PIK3CA**	46%	37%
**CYR61**	15%	15%		**SCUBE2**	36%	36%
**EGFR**	34%	34%		**TIMP1**	20%	20%
**ERBB3**	40%	36%		**TIMP3**	65%	65%
**ERBB4**	33%	33%		**TK1**	67%	52%
**ESR1**	45%	45%		**TNFRSF10B**	12%	12%
**FN1**	34%	34%		**VEGFA**	28%	23%
**GDF15**	114%	68%		**WT1**	69%	45%
**IGF2**	45%	45%		**YWHAZ**	16%	16%
**LCN2**	47%	34%				

Table S5: *Percent CV values of original and unfiltered OpenArray PCR results. Percent CV values for all tissues (%CV a) and for only those tissues with outliers were removed from the OpenArray results (%CV b). Removal was acceptable because the OpenArray PCR was not duplicated like the original PCR and had a higher number of values close or below the PCR's detection limit.*
