# Supplementary material for: Comparison of Breast Cancer to Healthy Control Tissue Discovers Novel Markers with Potential for Prognosis and Early Detection
Source: PLoS One. 2010 Feb 9;5(2):e9122. doi: 10.1371/journal.pone.0009122 (PMC2817747; doi:10.1371/journal.pone.0009122)
Supplement: Table S6 — Composition of the Sub-Cluster of the Cancer Cluster. Shown are the number of patients and the percentage of the total number of patients per cluster. Highlighted are the features that give the patients in the luminal-enriched cluster a better prognosis. (0.03 MB PDF) [file pone.0009122.s009.pdf]

	basal-enriched		luminal-enriched	
**HISTOLOGY**				
**ductal**	16	80%	5	45%
**lobular, ductal-lobular**	1	5%	6	55%
**other**	3	15%	0	0%
**HR/HER2 STATUS**				
**HER2 negative, HR negative**	7	35%	0	0%
**HER2 positive, HR negative**	5	25%	0	0%
**HER2 positive, HR positive**	2	10%	2	18%
**HER2 negative, HR positive**	5	25%	8	73%
**N/A**	1	5%	1	9%
**PATIENT STATUS**				
**deceased**	5	25%	0	0%
**recurred**	6	30%	0	0%
**alive, NED**	9	45%	11	100%
**31 patients**	20	100%	11	100%

***Table S6**: Composition of the Sub-Cluster of the Cancer Cluster. Shown are the number of patients and the percentage of the total number of patients per cluster. Highlighted are the features that give the patients in the luminal-enriched cluster a better prognosis.*
