# Supplementary material for: Comparison of Breast Cancer to Healthy Control Tissue Discovers Novel Markers with Potential for Prognosis and Early Detection
Source: PLoS One. 2010 Feb 9;5(2):e9122. doi: 10.1371/journal.pone.0009122 (PMC2817747; doi:10.1371/journal.pone.0009122)
Supplement: Figure S1 — Unsupervised cluster analysis of the filtered OpenArray data (112 genes, 13 invasive cancers and 9 normal control tissues). The OpenArray dataset contains a high proportion of genes with under-expression in the invasive cancer tissues (pink shading on the left) compared to the healthy controls (green shading on the left). The genes found to be under- or over-expressed by the original dataset are indicated by a green or red dot next to their names (right). These are found in the expected cluster, further confirming the original PCR results. The tissue descriptor above lists the histology (IDC in pink, ILC and IDLC in orange and NML in green) followed by the tissue identifier. (0.23 MB PDF) [file pone.0009122.s010.pdf]

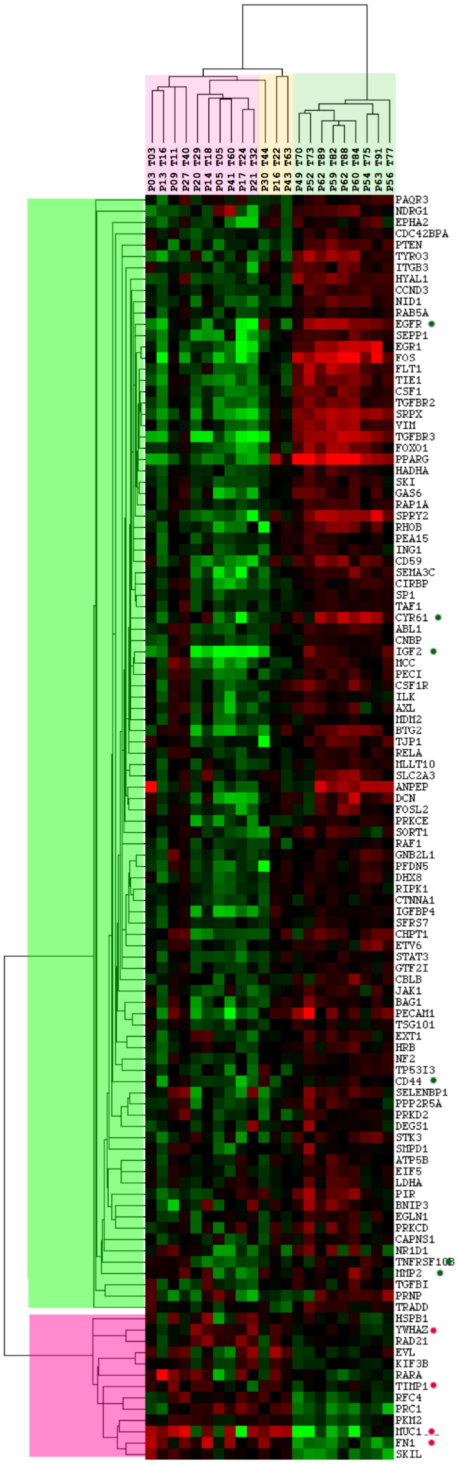

Figure S1: *Unsupervised cluster analysis of the filtered OpenArray data (112 genes, 13 invasive cancers and 9 normal control tissues). The OpenArray dataset contains a high proportion of genes with under-expression in the invasive cancer tissues (pink shading on the left) compared to the healthy controls (green shading on the left). The genes found to be under- or over-expressed by the original dataset are indicated by a green or red dot next to their names (right). These are found in the expected cluster, further confirming the original PCR results. The tissue descriptor above lists the histology (IDC in pink, ILC and IDLC in orange and NML in green) followed by the tissue identifier.*
