# Supplementary material for: Comparison of Breast Cancer to Healthy Control Tissue Discovers Novel Markers with Potential for Prognosis and Early Detection
Source: PLoS One. 2010 Feb 9;5(2):e9122. doi: 10.1371/journal.pone.0009122 (PMC2817747; doi:10.1371/journal.pone.0009122)
Supplement: Figure S2 — Unsupervised cluster analysis of the 24 invasive cancer tissues (7 tissues with > = 60% and 17 with < = 30% viable tumor cells) using the 67 genes that discriminate between invasive tissues and mam- maplasty normal tissues. No cluster formation is observed based on tumor cell content. The two clusters that are formed separate the cancers by outcome. (0.24 MB PDF) [file pone.0009122.s011.pdf]

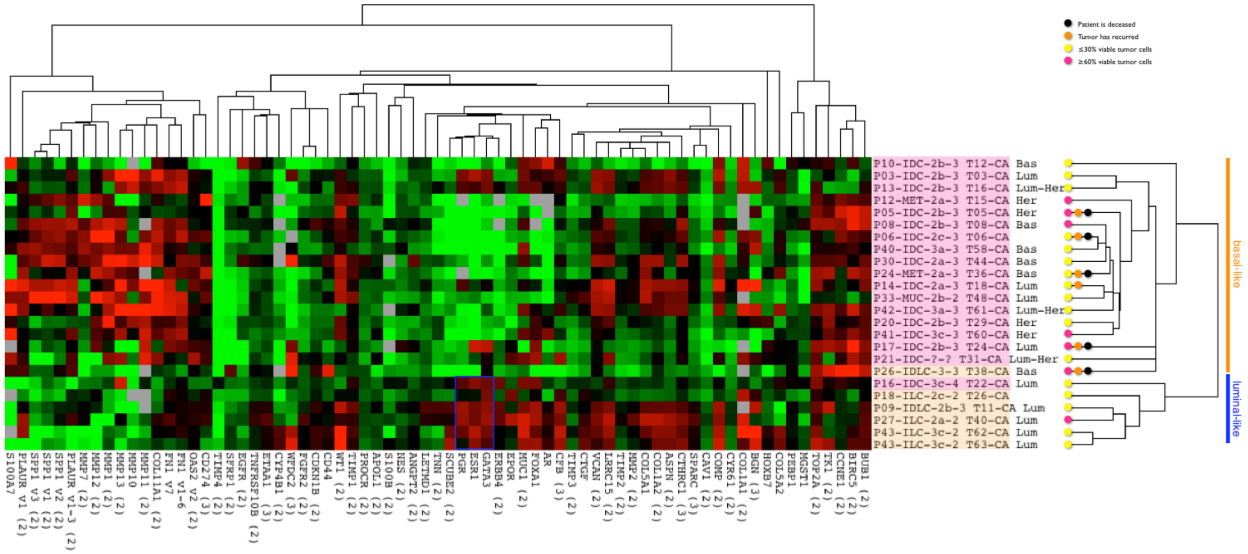

Figure S2: *Unsupervised cluster analysis of the 24 invasive cancer tissues (7 tissues with ≥60% and 17 with ≤30% viable tumor cells) using the 67 genes that discriminate between invasive tissues and mammaplasty normal tissues. No cluster formation is observed based on tumor cell content. The two clusters that are formed separate the cancers by outcome.*
