# Supplementary figures and images for: Correction: Comparison of Breast Cancer to Healthy Control Tissue Discovers Novel Markers with Potential for Prognosis and Early Detection
Source: PLoS One. 2010 Apr 2;5(4):10.1371/annotation/632c5ae8-271b-4d19-8509-dc3b2eefe6a4. doi: 10.1371/annotation/632c5ae8-271b-4d19-8509-dc3b2eefe6a4 (PMC2849775; doi:10.1371/annotation/632c5ae8-271b-4d19-8509-dc3b2eefe6a4)

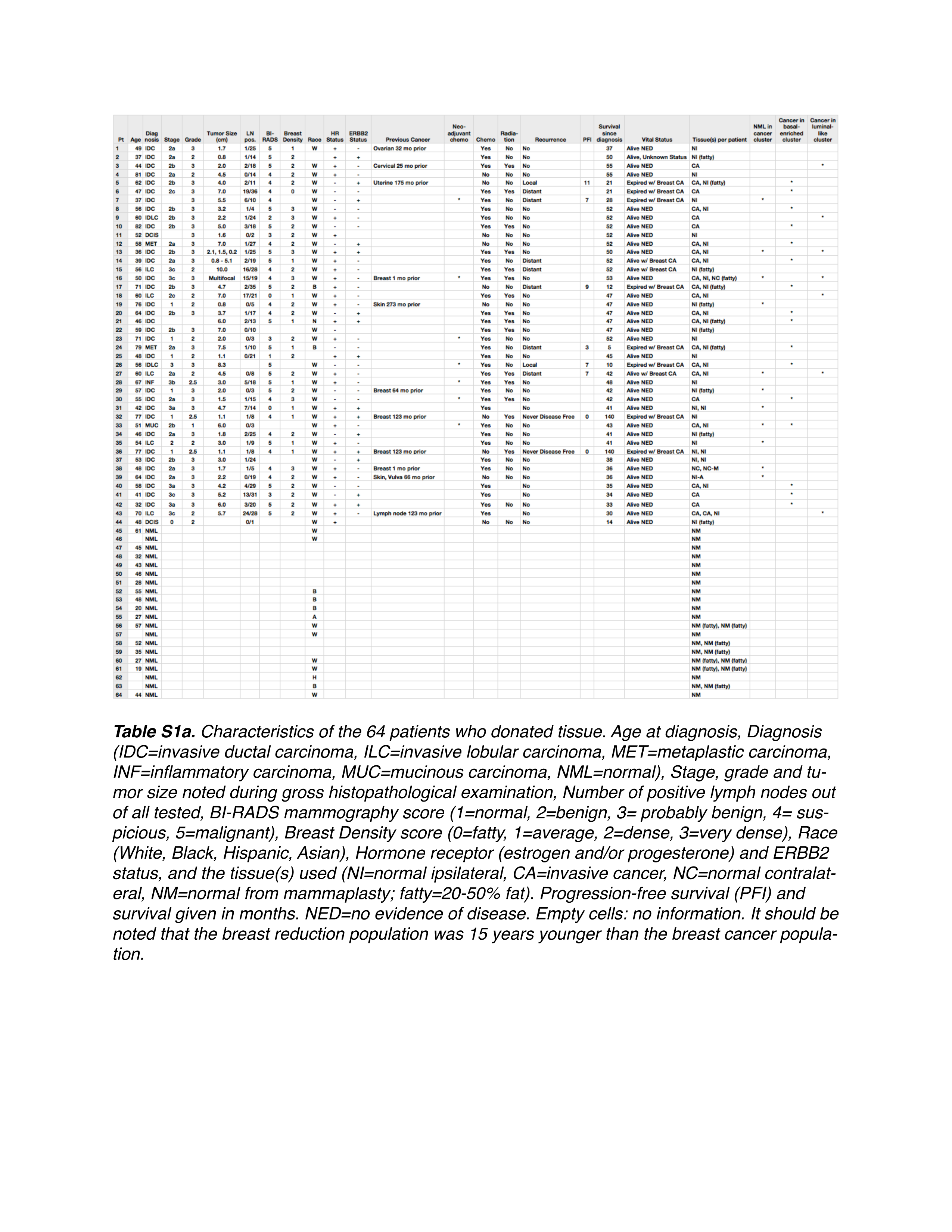

Supplement: Supplementary file 1 [file pone.632c5ae8-271b-4d19-8509-dc3b2eefe6a4.s001.tif]
